# Coniferaldehyde reverses 3-nitropropionic acid-induced Huntington’s disease pathologies via PKM2 restoration and JAK2/STAT3 inhibition

**DOI:** 10.1186/s10020-025-01308-0

**Published:** 2025-07-31

**Authors:** Ayooluwa Gabriel Ibiayo, Peeraporn Varinthra, Mukundan Nagarajan, Ingrid Y Liu

**Affiliations:** 1 Institute of Medical Sciences, College of Medicine, Tzu Chi University, Hualien City, 97004 Taiwan; 2 Department of Molecular Biology and Human Genetics, College of Medicine, Tzu Chi University, Hualien City, 97004 Taiwan

**Keywords:** Coniferaldehyde, Huntington’s disease, JAK2/STAT3 signaling, PKM2, Oxidative stress, Neuroinflammation

## Abstract

**Background:**

Huntington’s disease (HD) is a fatal neurodegenerative disorder characterized by progressive motor decline and neuronal loss, with no curative disease-modifying therapies available. The mitochondrial toxin 3-nitropropionic acid (3-NP) is widely used to model HD-like pathologies. We investigated the therapeutic potential of coniferaldehyde (CFA), a natural phenolic compound with anti-inflammatory, antioxidant, and anti-radical properties, against 3-NP-induced neurodegeneration. Given the roles of oxidative stress, metabolic dysfunction, and neuroinflammation in HD, we hypothesize that CFA exerts neuroprotection by attenuating these processes via the Janus kinase 2/signal transducer and activator of transcription 3 (JAK2/STAT3) pathway - a novel target for CFA in HD.

**Methods:**

Neurological and behavioral deficits were assessed via neurological assessment scaling, rotarod, and open field tests. Nissl staining was performed to evaluate neuronal damage in the motor cortex and striatum. Dihydroethidium staining (DHE) was used to measure reactive oxygen species (ROS) levels, and the terminal deoxynucleotidyl transferase-mediated dUTP nick end labeling (TUNEL) assay was conducted to detect apoptosis. Western blot assay and immunofluorescence staining were used to examine CFA’s effect. Additionally, molecular docking was performed to analyze CFA’s interaction with STAT3.

**Results:**

CFA treatment significantly improved motor function, preserved neuronal architecture, and reduced apoptosis, as confirmed by Nissl and TUNEL staining. CFA also decreased ROS levels and restored pyruvate kinase M2 (PKM2) expression, a key regulator of metabolic homeostasis. Consistently, CFA attenuated neuroinflammation by suppressing Glial Fibrillary Acidic Protein (GFAP) expression and proinflammatory cytokines Interleukin-6 (IL-6) and Interleukin-1 beta (IL-1β). Molecular docking studies revealed a strong binding affinity between CFA and STAT3, and western blot analysis showed reduced phosphorylation of STAT3, indicating modulation of the JAK2/STAT3 signaling pathway.

**Conclusion:**

These findings demonstrate that CFA modulates oxidative, PKM2-mediated metabolic, and inflammatory pathways through the JAK2/STAT3 axis, enhancing motor function and neuronal survival in a 3-NP model of HD. This multi-targeted mechanism highlights its potential as a disease-modifying therapy for advancing therapeutic strategies in HD and related neurodegenerative disorders.

**Graphical abstract:**

Neuroprotective mechanism of CFA in a 3-NP-induced HD model. 3-NP induces HD-like pathology in the motor cortex and striatum by inhibiting succinate dehydrogenase (Complex II), leading to ATP depletion, increased reactive oxygen species (ROS), neuroinflammation, apoptosis, PKM2 dysregulation, neurological impairments, and motor deficits. CFA treatment attenuates these pathological processes by reducing ROS and apoptosis, restoring PKM2 expression, and modulating glial activation and proinflammatory cytokines (IL-6 and IL-1β). In silico docking and in vivo analyses further show that CFA reduces phosphorylation of STAT3, suggesting suppression of the JAK2/STAT3 pathway as a key mechanism of action. CFA promotes neurological and motor improvement, metabolic, and inflammatory homeostasis, supporting its potential as a disease-modifying therapeutic for HD.
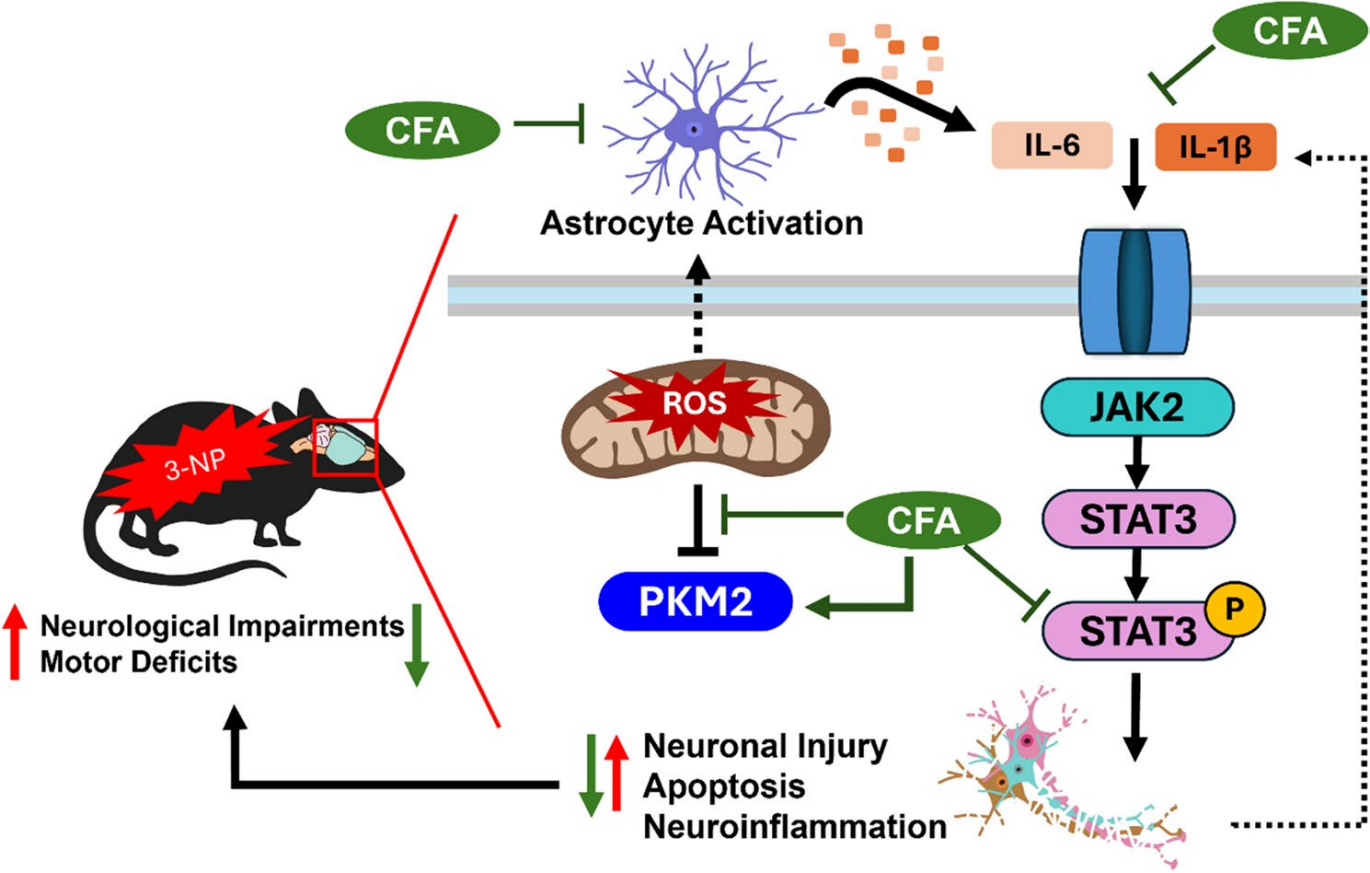

**Supplementary Information:**

The online version contains supplementary material available at 10.1186/s10020-025-01308-0.

## Background

Huntington’s disease (HD) is a progressive, fatal neuronal degeneration that impairs motor function and cognition and presents psychiatric symptoms (Saade and Mestre 2024). It arises from a mutation in the huntingtin gene, accumulating mutant huntingtin protein (mHTT), and subsequent neuronal damage, particularly in the striatum and cortex (A novel gene 1993). HD pathophysiology involves complex mechanisms, including oxidative stress, mitochondrial dysfunction, neuroinflammation, and apoptosis, culminating in neuronal loss in these regions. Excessive reactive oxygen species (ROS) exacerbate neuroinflammation, apoptosis, and neuronal damage (Podlacha et al. 2022). With no cure available and current treatments limited to symptom management, identifying novel therapeutic targets remains an urgent priority (Singh et al. 2024).

The 3-nitropropionic acid (3-NP) model is widely used to replicate HD-like neurodegeneration, inducing motor deficits, mitochondrial dysfunction, oxidative stress, neuroinflammation, and corticostriatal cell death, key hallmarks of HD (Ludolph et al. 1991; Brouillet et al. 2005; Cantero-Tellez et al. 2025; Kim et al. 2017a). The IL-6/JAK2/STAT3 signaling pathway, critical for regulating neuroinflammation and neuronal survival, is dysregulated in neurodegenerative conditions like HD (Ben Haim et al. 2015; Kooshki et al. 2023; Panda et al. 2024). Inflammatory processes marked by elevated plasma IL-6 levels may begin up to 16 years before the appearance of clinical symptoms (Bjorkqvist et al. 2008). Similarly, aberrant upregulation of IL-1β has been linked to HD pathogenesis (Noureldeen et al. 2024). In HD, neuronal loss is accompanied by increased astrocyte reactivity, marked by GFAP upregulation (Elbaz et al. 2025), and impaired astrocytic functions such as ion balance, Ca^2+^ signaling, and neurotransmitter clearance, all contributing to disease progression (Brown et al. 2023). Notably, upregulation of the astrocytic-IL6/JAK2/STAT3 pathway promotes a pro-inflammatory response and neuronal apoptosis, making it a promising therapeutic target (Ben Haim et al. 2015; Yin et al. 2018; Crotti and Glass 2015). Energy metabolism and dysregulation in homeostatic balance have been shown to contribute to the pathology of HD (Nasr et al. 2003; Adanyeguh et al. 2015). PKM2 (pyruvate kinase M2) is a multifaceted glycolytic enzyme with crucial roles in energy metabolism, cell proliferation, differentiation, and survival (He et al. 2025). Its expression and activity are tightly regulated, and it plays significant roles in the pathogenesis of various neurological diseases, including Alzheimer’s disease (Han et al. 2021), Ischemic stroke (Chen et al. 2018), as well as in normal brain functions like neurogenesis and cognition (He et al. 2025). Interestingly, the role of PKM2 under 3-NP-HD-like pathology remains largely unknown. Considering the roles of oxidative stress and neuroinflammation in HD, targeting PKM2 and modulating the IL-6/JAK2/STAT3 pathway with a multifaceted therapeutic may offer a novel strategy to mitigate disease progression.

Lignin, a complex biopolymer abundant in plant cell walls, is a rich source of phenolic compounds with demonstrated bioactivity (Renault et al. 2019). Coniferaldehyde (CFA), a lignin-derived natural phenolic compound derived from dietary and medicinal plants (Chen et al. 2018; Renault et al. 2019; Dong et al. 2020; Akram et al. 2016), exhibits antioxidant and anti-inflammatory properties in various disease models (Dong et al. 2020; Akram et al. 2016; Kim et al. 2016; Park et al. 2024). However, its potential to modulate HD-related neuropathology mainly through regulating ROS, apoptosis, and neuroinflammation remains unexplored. Computational approaches, such as molecular docking and dynamic simulations, have proven valuable in elucidating ligand-protein interactions to identify potential drug candidates (Liu and Cao 2024).

This research investigates the therapeutic potential of CFA in a 3-NP-induced HD mouse model. The study examines whether CFA can reduce ROS generation, prevent neuronal loss, decrease apoptosis (via fewer TUNEL-positive cells), improve PKM2 energy metabolism, and suppress neuroinflammation through the IL-6/JAK2/STAT3 pathway. Molecular docking and dynamic simulations explored CFA’s interaction with STAT3. By deciphering the molecular pathways through which CFA exerts its neuroprotective effects, the study aims to lay the groundwork for developing CFA as a new treatment strategy for HD and other neurodegenerative conditions.

## Materials and methods

### Animals

Eight-week-old male C57BL/6J wild-type mice were obtained from the National Laboratory Animal Centre (Taiwan) and housed individually at Tzu Chi University’s Laboratory Animal Centre under standardized conditions (12-hour light/dark cycle, with ad libitum access to food and water) until behavioral tasks were conducted.

### Experimental procedures

#### Experimental groups

Fifty-five male mice were used for this study. All mice were housed individually in their cages for 5 days of acclimatization; thereafter, the animals were randomly assigned to control and treatment groups. Group 1 (Vehicle) received Saline and DMSO or saline alone, Group 2 (Model) received DMSO and 3-NP, Group 3 received CFA 10 mg/kg, and 3-NP, Group 4 received CFA 20 mg/kg and 3-NP, Group 5 received CFA 20 mg/kg only. The behavioral experiments were performed in a quiet room during the light cycle phase.

#### 3-Nitropropionic acid (3-NP) intoxication and CFA administration

3-NP and CFA were purchased from Sigma (3-NP, N5636; CFA-382051 (98%), Sigma-Aldrich Chemical Co., MO, USA). 3-NP was dissolved in saline to a concentration of 13.2 mg/mL for 60 mg/kg and 17.6 mg/mL for 80 mg/kg (pH 7.4). Solutions were filtered through a 0.2 μm filter under a laminar flow hood to remove any bacteria before being stored in the dark at 4˚C until further use. Two stock solutions were prepared to optimize dosing accuracy, maintain solubility, and minimize dilution errors or contamination risk. The 3-NP solution was administered via the intraperitoneal region (*i.p.*) twice daily for 2 days at 12-hour intervals (7:00 a.m. and 7:00 p.m.). We consistently performed the experiments during the 12 h light cycle at the same time of day. To enable the use of fewer animals while achieving reproducible significant HD-like pathologies, and to determine the potential neuroprotective effect of CFA targeting cortico-striatal damage, and defects in energy metabolism, the dosage 60 mg/kg on the first day and 80 mg/kg on the second day (60-60-80-80 dose regimen) was utilized. The 3-NP intoxication schedule is predicated on prior studies demonstrating striatal neurotoxicity (Kim et al. 2017a; Ahmed et al. 2021; Yang et al. 2021; Liu et al. 2018). CFA was dissolved in saline: dimethylsulfoxide (DMSO) at a ratio of 9:1 and administered *i.p.* for six days before 3-NP intoxication, followed by an additional two days of treatment, beginning 24 h after the final 3-NP administration. The experimental schedule is in Supplementary Section Figure S1. Based on previous studies (Akram et al. 2016; Jeon et al. 2019) and pilot study results (refer to Supplementary Section Figure S2 and S3), we have selected the dose of CFA 20 mg/kg for *i.p.* administration as this dose was efficacious. The rationale for the study design was adapted from previous studies with a slight modification (Jeon et al. 2019; Silva-Palacios et al. 2017).

### Drug metabolism and pharmacokinetics (DMPK) analyses of CFA

The SwissADME algorithm (Daina et al. 2017) was employed to evaluate the DMPK profile, ADMET characteristics, and pharmacological properties of CFA. As summarized in Supplementary Tables S1-S3, CFA exhibits prominent biological features. Additionally, the pharmacokinetic properties and drug-likeness of CFA were assessed in comparison to Curcumin (CUR), Resveratrol (RES), and Chlorogenic acid (CGA) using two established in silico tools: SwissADME (Daina et al. 2017) and ProTox 3.0 (Banerjee et al. 2024). Parameters evaluated included organ toxicity, molecular weight, LogP, aqueous solubility, gastrointestinal (GI) absorption, blood-brain barrier (BBB) permeability, CYP450 enzyme inhibition, and compliance with drug-likeness rules (Lipinski and Veber criteria). Predictions were based on canonical SMILES input, and comparative analysis was performed to contextualize CFA’s CNS-targeted pharmacokinetic profile among structurally related phenolic compounds. (See supplementary Tables S2 and S3).

### Neurological behavioral semi-quantitative assessment

We assessed the 3-NP-induced motor deficits using a behavioral semi-quantitative procedure (Fernagut et al. 2002; Jang et al. 2014). The motor behavior paradigm employs a three-level scale (0, 1, and 2) to quantify the extent of damage caused by 3-NP intoxication (maximum score = 10). The motor behavior scale for evaluating the severity of 3-NP-induced motor impairments in mice is rated 0 = no detectable neurological deficit (N.D.), 1 = mild deficit, and 2 = severe deficit. The following neurological parameters for significant motor deficit symptoms were observed: Hindlimb clasping, general locomotor activity, truncal dystonia, hindlimb dystonia, and postural challenge (compromised postural correction following the postural challenge - when the mouse is tipped onto its side, the animal fails to right itself and cannot return to an upright stance unaided). The 3-NP-induced clinical signs (Huang et al. 2006) and severity score are detailed in Supplementary Table S4. A digital video camera recorder (SONY, Japan) was used to record the assessment. The assessments were undertaken blinded to the conditions.

### Rotarod test

The Rotarod Test assessed motor coordination in mice. Using a previously described protocol (Lin et al. 2015; Yang et al. 2023), after acclimating to the sound of the rotarod for 15 min, mice underwent a training session consisting of three 60-second trials on the rod, starting at 0 rpm for 60 s and then 4 RPM for 60 s, with 10-minute intervals between trials. After a 30-minute rest, the test trial began, where mice were subjected to three test trials on the rod, starting at 4 RPM and increasing to 40 RPM over 300 s per trial, with 15-minute intervals between trials. Subsequently, at the inter-trial intervals, the mice were allowed to rest in their home cages, and the rotarod apparatus was cleaned with 70% Ethanol and wiped with paper tissue to remove odour cues, urine, and fecal discharge from the mice. The average latency to fall (when a mouse cannot stay upright on the rotating rod and drops to the trip-box) from the three test trials was recorded as the outcome measure. The Rotarod machine (Ugo Basile Mouse Rotarod, #Cat. No. 47650, Ugo Basile SRL, Gemonio, Italy) was used to evaluate the motor coordination of the animals, where, under normal conditions, a healthy mouse should spend more time on the rotating rod.

### Open field test

Using the previously published protocol, we used the Open Field Test to assess the rodents’ locomotor activity and exploratory behavior (Yang et al. 2021; Fernagut et al. 2002; Rastoldo et al. 2020). The test was conducted in a nontransparent plastic cage 50 cm (H) x 50 cm (L) x 50 cm (W) chamber with a white base and black walls under dim light. Using the Ethovision tracking software, a 50 cm calibration was applied for the arena settings. Next, the chamber was divided into two zones (corner and middle). The corner zones were marked as (S1-S6, S10-11, S15-16, S20-25), while the middle zone as (S7-S9, S12-S14, S17-S19). The animals were allowed to explore the entire open field freely. The chamber was cleaned with 70% ethanol between trials to remove any odour cues, urine, and feces of the animals. Using EthoVision for tracking, we employed the dynamic subtraction approach (Rastoldo et al. 2020) at a fixed sampling rate of 29.97 Hz. EthoVision automatically tracked each animal’s nose, center, and tail base throughout the recordings. We used a single analysis profile for data processing and focused on four dependent variables. The video capturing and tracking system (EthoVision XT 15) was used to record and analyze each animal’s total distance moved, total speed, total immobility, and mobility time within the entire chamber for 5 min (Yang et al. 2021; Fernagut et al. 2002). For mobility state, using the previously published method (Rastoldo et al. 2020) with slight modification the mobility threshold (the numeric cutoff that distinguishes “mobile” versus “immobile” (or resting) states in the EthoVision software) duration mobile and immobile were calculated with an average interval of 1 sample and a threshold for mobile between 5 and 60% and immobile below 5% (Rastoldo et al. 2020).

### Nissl staining

NeuroTrace™ 435/455 blue fluorescent Nissl stain (Cat #N-21479, Molecular Probes) was applied to brain sections following the manufacturer’s protocol. Cardiac perfusion was performed by first perfusing the mice with 0.9% sodium chloride (NaCl) in double-distilled water (ddH_2_O), followed by 4% paraformaldehyde (PFA). After extraction, the brains were immediately fixed in cold PFA (4°C) for 24 h, then transferred to 30% sucrose at 4°C for cryoprotection. Brain tissues were sectioned into 30 μm coronal slices using a cryostat (Leica CM3050S, Leica Biosystems, Nussloch GmbH). Sections were selected from bregma points 1.10 mm to 0.14 mm and washed three times in 1X phosphate-buffered saline (PBS) for 10 min each, then permeabilized in PBS containing 0.1% Triton X-100 for 10 min at room temperature (RT). After two additional 5-minute washes in PBS, the sections were incubated with NeuroTrace stain (1:200 in PBS) for 20 min at RT in the dark with gentle shaking. The stain was removed, and sections were washed in PBS with 0.1% Triton X-100 for 10 min, followed by two 5-minute washes in PBS. Sections were washed overnight at 4°C in PBS, mounted, and cover-slipped. Images were captured from the motor cortex, dorsomedial (dm), dorsolateral (dl), centromedial (cm), and centrolateral (cl) regions of the striatum (Brown et al. 2023) using a NIKON C2si + confocal microscope (Nikon, Japan). The mean fluorescence intensity analysis was performed with ImageJ version 1.53k (National Institute of Health (NIH), USA).

### Detection of reactive oxygen species (ROS) levels

To assess ROS levels in the motor cortex and striatum, mice were sacrificed, and brains were isolated following trans-cardiac perfusion, then fixed in 4% PFA overnight at 4°C. The brains were subsequently stored in 30% sucrose at 4°C. The procedure followed a previous study (Phasuk et al. 2021). Fixed brains were sliced at 30 μm thickness using a cryostat and temporarily stored in cryoprotectant at −20°C. Sections were washed in PBS (three times, 10 min each) and immersed in 1 µM/ml dihydroethidium (DHE, Invitrogen) in PBS at RT for 5 min in the dark to determine intracellular hydrogen peroxide (H_2_O_2_). After immersion, sections were washed with PBS, mounted on slides, and cover-slipped. DHE oxidizes to ethidium by superoxide anions, which bind to nuclear DNA, emitting red fluorescence. Images from the motor cortex, dm, dl, cm, and cl regions of the striatum were captured using a NIKON C2si + confocal microscope at 561 nm with a 20x objective lens and analyzed with ImageJ version 1.53k (NIH, USA).

### Immunofluorescence staining

For immunofluorescence staining, the mice were anesthetized and transcardially perfused, starting with 0.9% saline, then followed by 4% PFA. Brains were stored in cold PFA at 4°C for 24 h and then transferred into 30% sucrose at 4°C. Using a cryostat (Leica CM3050S, Leica Biosystems Nussloch GmbH), 30 μm sections were sliced and stored in a cryoprotectant solution (200 mM phosphate buffer, 62.5 ml, Sucrose 75 g, Ethylene glycol 75 ml, and Polyvinylpyrrolidone PVP-40 2.5 g + 250 ml of ddH_2_O) at −20°C. The sections were washed thrice, 10 min each in 1xPBS, then permeabilized in permeating buffer (1% Triton X-100 and 2% Tween 20 in PBS) for 30 min. After that, a blocking buffer (1% normal goat serum (NGS) and 0.3% Triton X-100 in PBS) was added to the blocking process at RT for 1 h. Next, primary antibodies against PKM2 (1:100; CST, USA), and IL-6 (1:100; Cell Signaling, #12912s) were applied and incubated overnight at 4°C. Sections were washed with 0.25% Triton in PBS (three times for 10 min each at RT), then incubated with corresponding secondary antibodies Alexa fluor 488 Goat anti-Rabbit, 1:300 directed against IL-6 or Alexa fluor 594, Goat anti-Rabbit, 1:300, directed against PKM2, (ThermoFisher, USA) for 1 h at RT. The sections were counterstained with DAPI (4̍,6-Diamidino-2-phenylindole) for 10 min, then washed in PBS three times, 10 min each. Sections were mounted on glass slides and covered with Fluoromount™ aqueous mounting medium (Sigma Aldrich, USA, F4680). Images from the motor cortex, dm, dl, cm, and cl regions of the striatum were captured using the NIKON C2si + confocal microscope under 405 nm, 488 nm, and 561 nm wavelengths with 20x objective lenses. Fluorescence-positive areas were analyzed using ImageJ version 1.53k (NIH, USA) from 3 to 4 sections per mouse.

### Immunoblotting analysis

The striatum was dissected for western blotting and placed in a lysis buffer containing 1X RIPA buffer (Millipore, USA) with protease and phosphatase inhibitors on ice. The tissue lysates were centrifuged at 13,500 rpm for 15 min at 4°C, and the supernatant was stored at −80°C. Protein concentration was determined using Bradford’s assay before samples were subjected to SDS-PAGE. Equal protein samples were loaded, separated by a 10% or 12% SDS-PAGE, and transferred onto the polyvinylidene fluoride (PVDF) membrane. Following the transfer, the membrane was blocked with 1% bovine serum albumin (BSA) in tris-buffered saline with Tween-20 (TBST) for 1 h at RT. Primary antibodies against GFAP (1:1000; Abcam, UK), PKM2 (1:1000; CST, USA, 4053 S), IL-6 (1:1000; Cell signaling, 12912), JAK2 (1:1000; Cell signaling, 3230 S), STAT3 (1:1000; Cell signaling, 4904 S), p-STAT3 (Tyr705) (1:1000; Cell signaling, 9131 S), IL-1β (1:1000; Abcam, 9722), β-actin (1:10,000; Sigma-Aldrich, USA) were diluted in TBST with 0.1% BSA incubated overnight at 4 °C. The membrane was washed three times for 10 min each, followed by incubation with the corresponding secondary antibody (Anti-mouse #7076S or Anti-Rabbit 7074 S, Cell signaling) in 0.1% BSA or TBST only for 1 h at RT. After three 10-minute washes with TBST, protein bands were developed using ECL (Western lighting^®^ Plus ECL, PerkinElmer Inc., MA, USA). Protein detection was conducted using the Thermo iBright™ CL1500 and FL1500 (A44241) Imaging Systems. For each biological replicate, at least two technical replicates (duplicate gels run on different days) were analyzed, and their normalized R values relative to the control were averaged to yield one R per biological replicate. All normalized R values (one per biological replicate per group) were used for the statistical analysis, and band intensities were quantified using ImageJ (NIH, version 1.53k).

### TUNEL assay

The TUNEL assay was performed according to the protocol in the TUNEL Assay Kit (Abcam, 66110). By employing the terminal deoxynucleotidyl transferase to label the deoxynucleotides with Br-dUTP (bromolated deoxyuridine triphosphate nucleotide) at the free 3’-hydroxyl ends of fragmented DNA, we carried out a TUNEL experiment using the (TdT) to catalyze the incorporation of the deoxynucleotides. Tissue sections were fixed with 4% PFA in PBS and then washed with fresh PBS. The sections were then treated with proteinase K in Tris-HCl (pH 8.0) with 50 mM EDTA at room RT for 5 min, briefly washed in PBS, and refixed with 4% PFA in PBS at RT for another 5 min. After washing, sections were incubated in DNA labeling solution for 60 min at 37°C, then washed again. They were subsequently incubated in antibody solution for 30 min at RT. The sections were counterstained with DAPI for 5 min and washed 3 times, 10 min each. The Confocal microscope was used for image analysis, with BrdU-Red labeling at Excitation/Emission = 488/576 nm. TUNEL- fluorescence intensity from the motor cortex, striatal dm, dl, cm, and cl regions were analyzed using ImageJ version 1.53k (NIH, USA) from 3 to 4 sections per mouse.

### Imaging analysis

Following a previously published method (Brown et al. 2023), Fluorescence intensities for each antibody (PKM2, IL-6), as well as Nissl, DHE, and TUNEL stains, were quantified in both the motor cortex and striatum as follows:

- Motor cortex: One full-field image per mouse (*n* = 3–5 mice/group), 3–4 coronal sections were acquired, converted to 8-bit, thresholded, and measured (mean gray value) in ImageJ. Each mouse contributed a single data point in the motor cortex plots, and group means ± SEM were calculated over the image values.
- Striatum: In each mouse (3–5 mice/group), 3–4 coronal sections were imaged; within each section, the four striatal subregions (dorsomedial (dm), dorsolateral (dl), centromedial (cm), centrolateral (cl)) were averaged to yield one “sectional striatum” value. All sectional values were plotted individually, and group means ± SEM were computed across these sectional values.

### Molecular docking

Molecular docking is a biological, computational method for studying the interaction between two molecules to determine their binding and stable complex formation. To predict the docking affinity and structural interaction, CBDOCK2, an online platform integrated with AUTODOCK VINA (Liu et al. 2022), was used for docking (PDB: 1BG1 retrieved from (https://www.rcsb.org/) with CFA, PubChem CID: 5280536 retrieved from (https://pubchem.ncbi.nlm.nih.gov/), and the binding score was used to predict the binding affinity. The full-length STAT3 monomer, chain A, was used in its entirety. It was prepared with USCF-CHIMERA X, with which the DNA-binding complex was removed alongside water molecules; the default settings were used for further peptide preparation. Additionally, for the identification of the binding cavity detection of STAT3 peptide, CBDOCK2 was utilized, and the peptide-ligand docking complexes, including the highest score complex, were obtained and visualized using Discovery Studio 2024 client tools.

### Statistical analysis

Data were initially tested for normality using the Shapiro-Wilk test. A Kruskal-Wallis test was employed for datasets that did not follow a normal distribution, followed by Dunn’s multiple comparisons and the Student T test for individual comparison. In contrast, normally distributed data were analyzed using a one-way ANOVA, followed by Tukey’s post hoc and Fisher’s LSD tests. Specifically, the Rotarod data were positively skewed; therefore, the values were log-transformed using the natural logarithm (Ln(Y)) to meet normality assumptions. Statistical significance was defined as *p* < 0.05. All data sets were analyzed with GraphPad Prism 9.0 (GraphPad Software, San Diego, CA, USA).

## Results

### CFA alleviates neurological impairment in 3-NP-injected mice

**Fig. 1:**
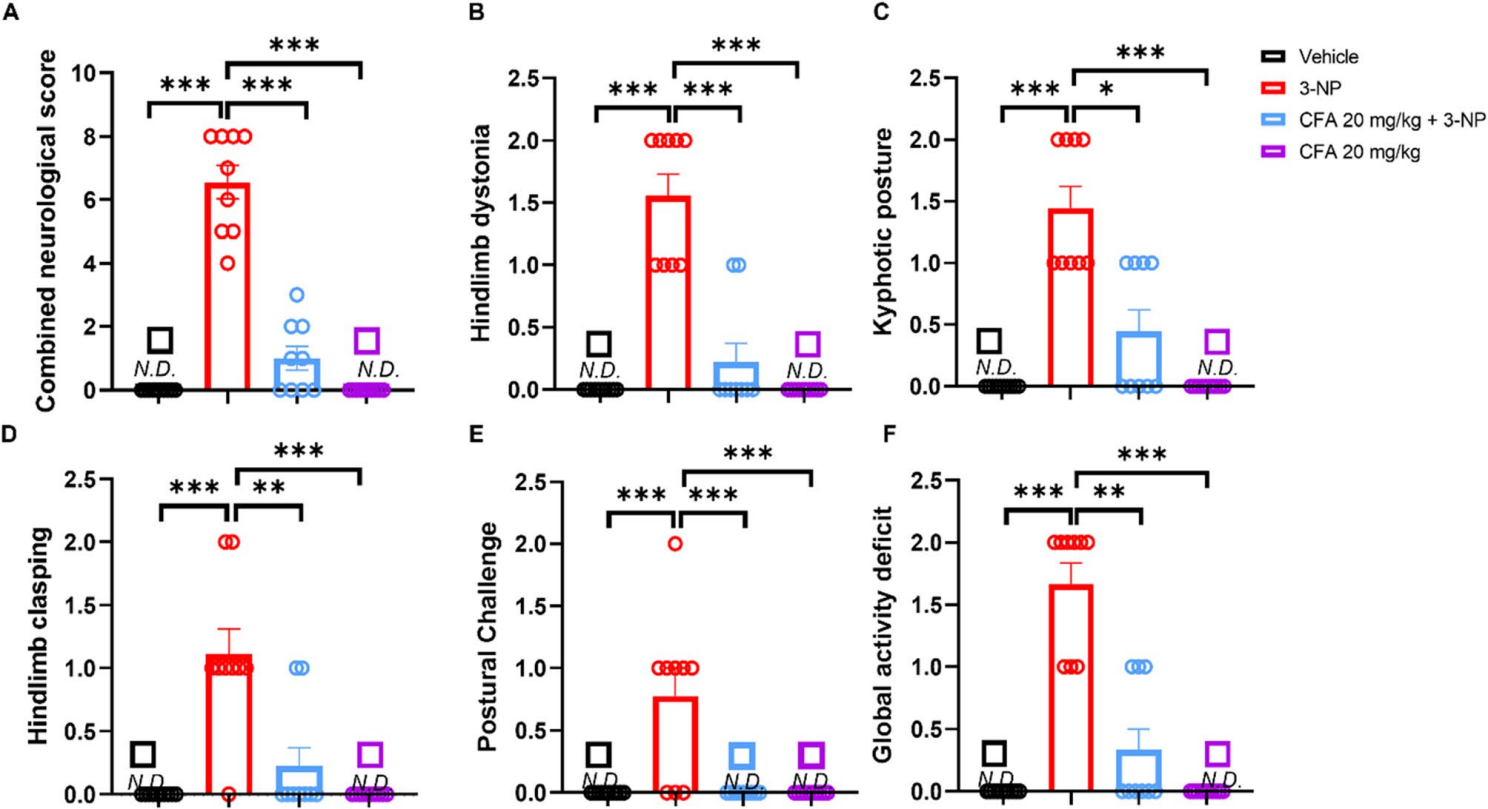
CFA alleviates 3-NP-induced neurological, motor, and abnormal postural phenotypes. The neurological assessment demonstrated **A** the neurological score, **B** hindlimb dystonia, **C** kyphotic posture, **D** hindlimb clasping, **E** postural challenge, and **F** global activity deficit in a free movement environment significantly impaired in the 3-NP group compared to vehicle and 20 mg/kg CFA only groups but significantly improved in CFA 20 mg/kg + 3-NP group. The data are expressed as mean ± SEM. The assessment scale is rated 0 = no detectable neurological deficit (N.D.), 1 = mild deficit, and 2 = severe deficit. One-way ANOVA followed by Tukey’s post hoc test was performed for **A**, and Dunn’s post hoc multiple comparisons for **B** to **F**. Significance level depicted as ***p < 0.001, **p < 0.01, and *p < 0.05. Vehicle, *n* = 10; 3-NP, *n* = 9; CFA+3NP, *n* = 9; CFA, *n* = 10

Here, we hypothesized that CFA may alleviate 3-NP-induced neurological deficits in mice. Our neurological assessment of the 3-NP group showed symptoms of severe neurological impairment, evidenced by a significantly elevated combined neurological score (*F*_(3,34)_ = 102.8, *p* < 0.001; Fig. 1A). This included hindlimb dystonia (*H*_(3)_ = 30.75, *p* < 0.001; Fig. 1B), truncal dystonia characterized by kyphotic posture (*H*_(3)_ = 28.50, *p* < 0.001; Fig. 1C), hindlimb clasping, (*H*_(3)_ = 25.07, *p* < 0.001; Fig. 1D), postural challenge, (*H*_(3)_ = 22.26, *p* < 0.001; Fig. 1E) and global activity deficit (*H*_(3)_ = 29.97, *p* < 0.001; Fig. 1F) in comparison to the Vehicle and CFA (20 mg/kg/day) group. The CFA 20 mg/kg + 3-NP mice displayed significantly decreased combined neurological scores (*p* < 0.001) compared to the 3-NP-only mice (Fig. 1A). Additionally, CFA 20 mg/kg + 3-NP mice significantly improved hindlimb dystonia (*p* < 0.001; Fig. 1B), truncal dystonia (*p* < 0.05; Fig. 1C), hindlimb clasping (*p* < 0.01; Fig. 1D), postural challenge (*p* < 0.001 Fig. 1E), and global activity deficit (*p* < 0.01; Fig. 1F) compared to the 3-NP-only mice. CFA 10 mg/kg + 3-NP group shows improvement in (A, *p* < 0.001), (B, *p* < 0.001), and (E, *p* < 0.01) only compared to the 3-NP-only group. (See Supplementary Fig. S2). Our findings indicate that CFA could alleviate neurological abnormalities of mice with 3-NP HD-like symptoms.

### CFA improved motor dysfunction in 3-NP-treated mice

**Fig. 2:**
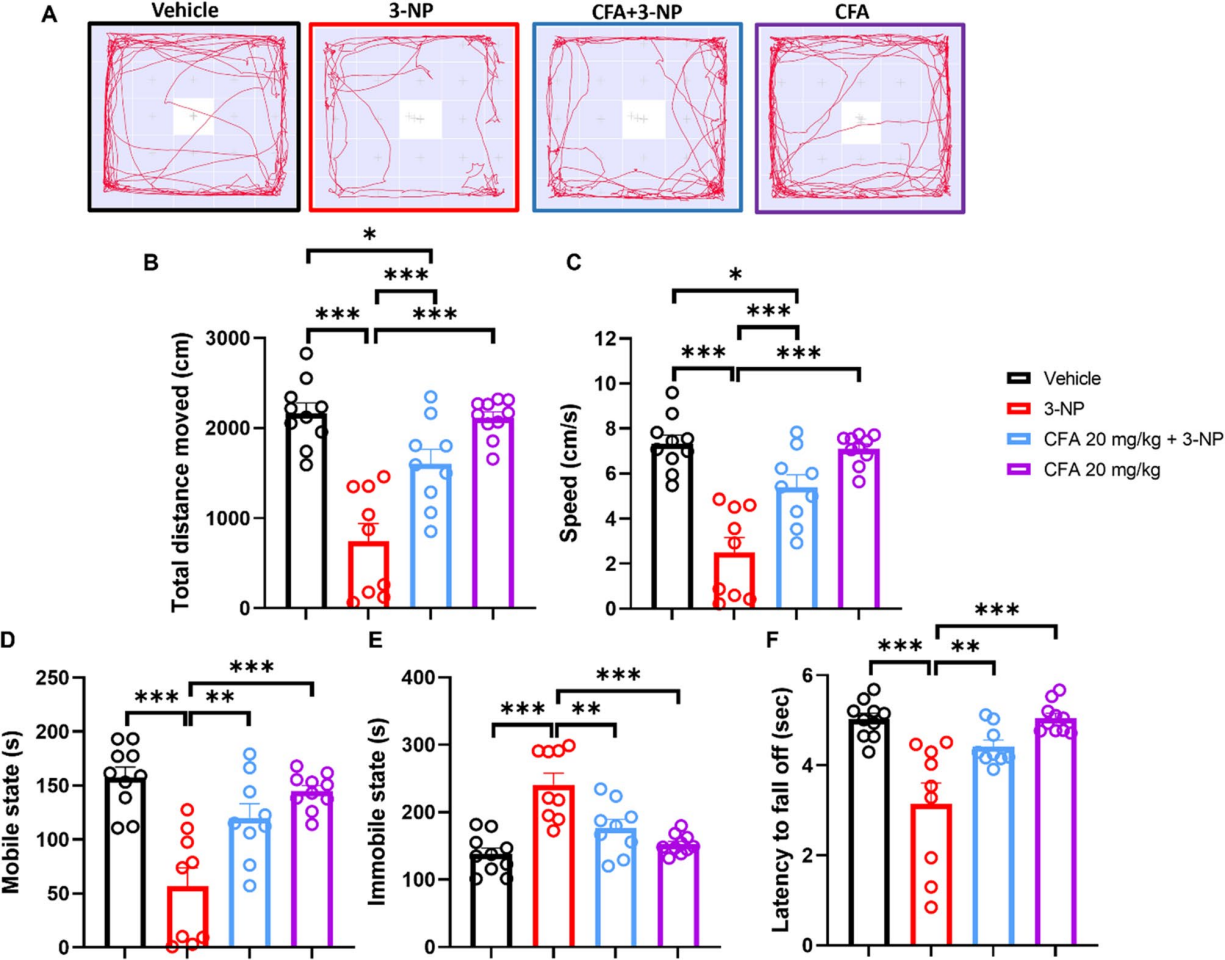
CFA significantly rescued 3-NP-induced locomotor and movement deficit behavior in HD mice. **A** OFT movement tracking images. The 3-NP group showed significantly decreased **B** total distance moved and **C** speed compared to vehicle and CFA-only groups, while significantly rescued after CFA 20 mg/kg administration. The 3-NP insult reduced **D** mobile state duration and increased **E** immobility state, but CFA relieved the mobility deficits. **F** Rotarod analysis shows that CFA treatment abated the motor coordination deficit caused by 3-NP. For **F**, the data are skewed positively, and a natural log (Y = Ln(Y)) transformation was applied to attain normality. The data are expressed as mean ± SEM. One-way ANOVA followed by Tukey’s post hoc test was performed for B to F. Significance level depicted as ***p < 0.001 **p < 0.01, and *p < 0.05. Vehicle, *n* = 10; 3-NP, *n* = 9; CFA + 3NP, *n* = 9; CFA, *n* = 10

Locomotor activity and motor coordination are used to assess the neurobehavioral function of mice. Using the open field and rotarod tests, we aimed to determine whether CFA may improve locomotor activity, exploration behavior, and motor coordination in the 3-NP mice. The mice were allowed to explore within the open field chamber for 5 min without disturbance. Movement tracking images were recorded (Fig. 2A). The results show that 3-NP mice have impaired locomotor activity, which caused a significant decrease in total distance moved (*F*_(3,34)_ = 21.88, *p* < 0.001; Fig. 2A), speed (*F*_(3,34)_ = 22.34, *p* < 0.001; Fig. 2B), and mobile state (*F*_(3,34)_ = 14.58, *p* < 0.001; Fig. 2C), and increase immobile state (*F*_(3,34)_ = 15.54, *p* < 0.001; Fig. 2D) compared to Vehicle (*p* < 0.001) and CFA 20 mg/kg-only (*p* < 0.001) groups. Interestingly, the CFA 20 mg/kg + 3-NP treatment group significantly enhanced the total distance moved (*p* < 0.001), speed (*p* < 0.001), and mobile state (*p* < 0.01) and reduced immobile state (*p* < 0.01) compared to the 3-NP group. However, the locomotor activity of the CFA 20 mg/kg + 3-NP showed partial improvement compared to the Vehicle group (*p* < 0.05). The CFA 10 mg/kg + 3-NP mice showed no effect on the locomotor activity in the open field test (See Supplementary Fig. S3A-E).

We further subjected the animals to the Rotarod test, evaluating the mice’s motor coordination. The Rotarod results revealed that 3-NP induced a motor coordination deficit in the mice (*F*_(3,34)_ = 12.76, *p* < 0.001; Fig. 2F) by decreasing the fall latency compared to the Vehicle (*p <* 0.001) and CFA 20 mg/kg-only (*p <* 0.001) groups, while CFA 20 mg/kg + 3-NP group increased the fall latency (*p* < 0.01). The CFA 10 mg/kg + 3-NP mice showed no rescue effect on the fall latency compared to the 3-NP-only group (See Supplementary Fig. S3A, F). This suggests that CFA mitigates locomotor impairments and motor dysfunction in the 3-NP-HD mice model.

### CFA confers neuroprotection against 3-NP-induced neuronal injury

**Fig. 3:**
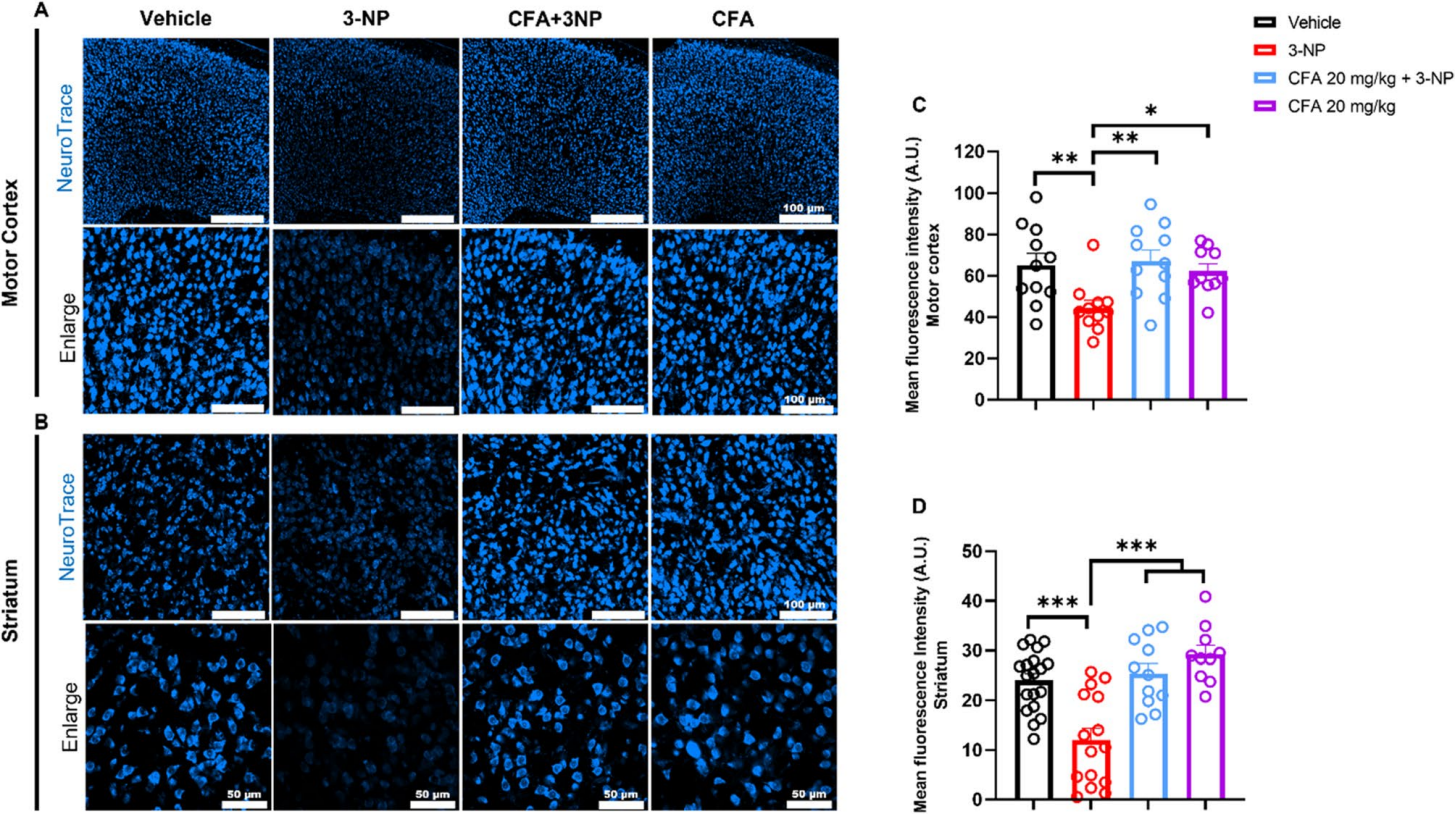
CFA exerts neuroprotection against 3–NP–induced neurodegeneration. Nissl granule fluorescence intensity in the **A** motor cortex and **B** striatum region showed reduced signal, signifying neuronal loss in both regions of the 3-NP group compared to the vehicle and CFA-only groups, but reversed in the CFA + 3-NP group. Data are shown for: the motor cortex: Vehicle, 3-NP, and CFA + 3-NP groups, each including *n* = 11 images from 3 mice; the CFA-only group includes *n* = 10 images from 3 mice. Striatum: Vehicle (*n* = 19 images from 5 mice), 3-NP (*n* = 15 images from 4 mice), and both CFA + 3-NP and CFA-only groups (*n* = 10 images from 3 mice each). Each dot represents the mean gray value of one full-field image, from 3–4 sections/mouse. The data are expressed as mean ± SEM. One-way ANOVA was applied, followed by Fisher’s (LSD*)* post hoc test for multiple comparisons. Significance level depicted as ^***^p < 0.001, ^**^p < 0.01, and ^*^p < 0.05. Scale bar = 100 μm (top panel) and 50 μm (enlarged image)

To determine whether CFA can prevent neuronal loss, we performed Nissl histological staining of the motor cortex and striatum brain tissue slices taken from bregma points 1.10 mm to 0.14 mm using the fluorescent NEUROTRACE, a selective marker for the Nissl substance in neurons (Madarasz et al. 2023; Min et al. 2024). The administration of 3-NP significantly reduced the fluorescent intensity in the 3-NP group, confirming neuronal loss and damage to the motor cortex (*F*_(3,39)_ = 5.052, *p* < 0.001; Fig. 3A and C) and striatal integrity (*F*_(3,52)_ = 15.57, *p* < 0.001; Fig. 3A and D) compared to the Vehicle group (*p* < 0.01 for Fig. 3C; *p* < 0.001 for Fig. 3D) and CFA-only groups (*p* < 0.05 for Fig. 3C; *p* < 0.001 for Fig. 3D). Notably, the CFA 20 mg/kg + 3-NP treatment groups significantly prevented the 3-NP-induced neuronal damage in the motor cortex (*p* < 0.01) and striatal regions (*p* < 0.001), indicating the neuroprotective potential of CFA.

### CFA abrogates 3-NP-induced apoptosis

**Fig. 4:**
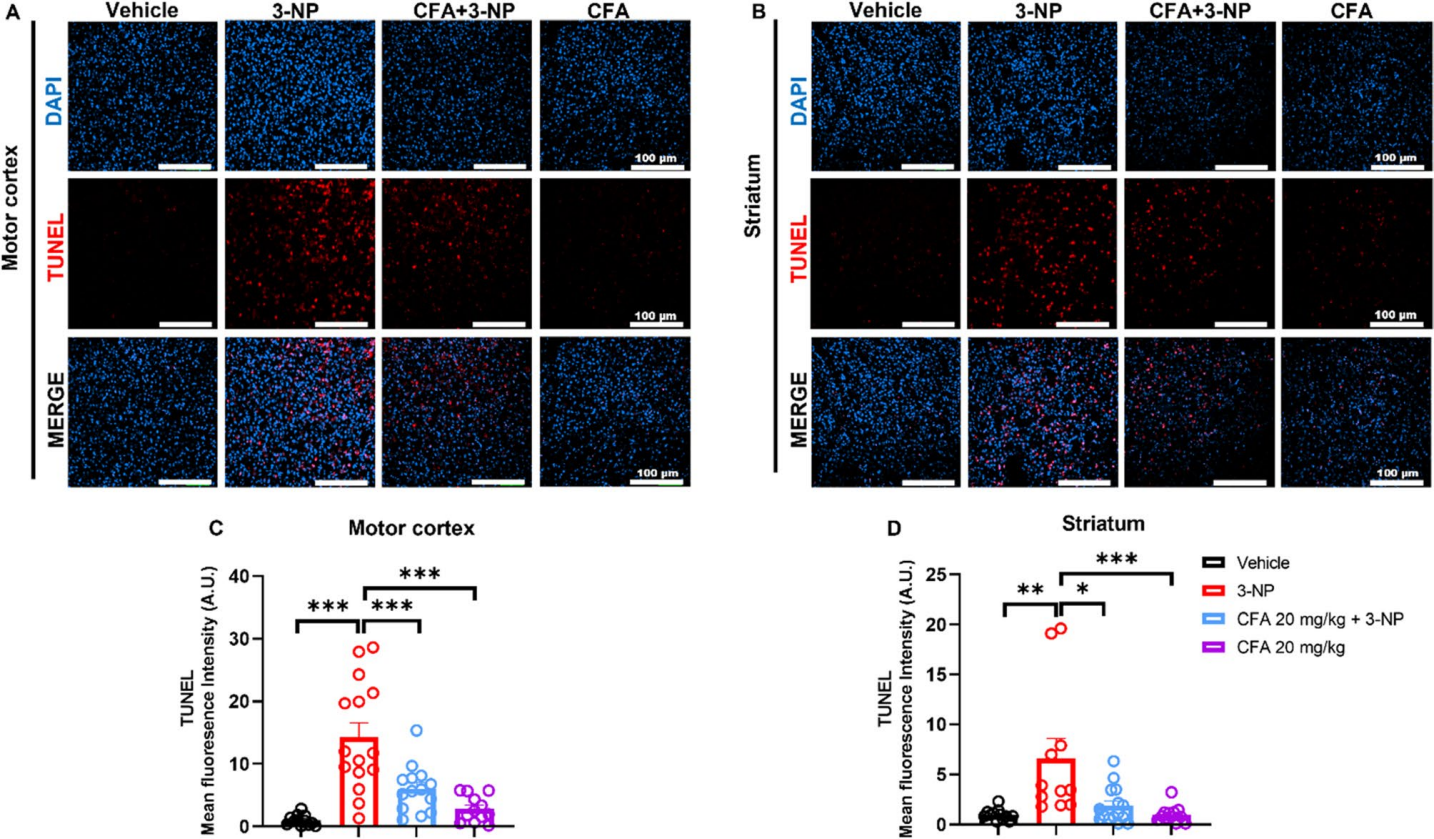
Anti-apoptotic effect of CFA against 3-NP-induced cortico-striatal apoptosis. Immunofluorescence images and quantitative results for the TUNEL fluorescence intensity signal in the **A**, **C** motor cortex and **B**, **D** striatum across groups. The results showed an elevated TUNEL signal in the 3-NP group compared to the Vehicle and CFA-only groups, while CFA reversed it in the CFA + 3-NP group. Data are shown for: Motor cortex: Vehicle (*n* = 12 images from 4 mice), 3-NP (*n* = 15 images from 5 mice), CFA + 3-NP (*n* = 15 images from 5 mice), CFA-only (*n* = 12 images from 4 mice). Striatum: Vehicle (*n* = 12 images from 4 mice), 3-NP (*n* = 11 images from 4 mice), CFA + 3-NP (*n* = 15 images from 5 mice), CFA-only (*n* = 12 images from 4 mice), from 3 sections/mouse. The data are expressed as mean ± SEM. **C** One-way ANOVA, followed by Tukey’s post hoc test, and **D** Kruskal-Wallis test, followed by Dunn’s post hoc test, were performed for multiple comparisons. Significance level depicted as ***p < 0.001 **p < 0.01, and *p < 0.05, scale bar = 100 μm

Subsequently, to determine whether neuronal cell death induced by 3-NP in Fig. 3 is apoptotic, a TUNEL assay was conducted. The results showed that 3-NP administration significantly induced apoptosis in the motor cortex (*F*_(3,49)_ = 18.11, *p* < 0.001; Fig. 4A, C) and striatum (*H*_(3)_ = 19.08, *p* < 0.001; Fig. 4B, D) compared to the Vehicle group (*p* < 0.001 for Fig. 4C; *p* < 0.01 for Fig. 4D) and CFA-only groups (*p* < 0.001 for Fig. 4C-D). Remarkably, CFA treatment significantly abrogated 3-NP-induced apoptosis in the motor cortex (*p* < 0.001) and striatum (*p* < 0.05), highlighting the anti-apoptotic potential of CFA.

### CFA reduces excessive ROS levels in 3-NP mice

**Fig. 5:**
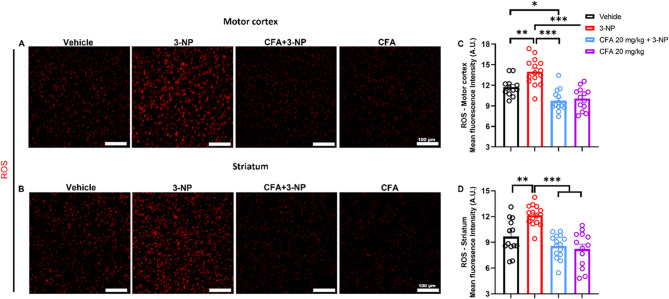
CFA attenuates elevated ROS levels in the 3-NP HD mice. The representative images for DHE-stained-ROS in **A** the motor cortex and **B** the striatum of all groups. **C** The motor cortex and **D** striatum of the 3-NP group showed elevated ROS expression compared to the vehicle and CFA-only group, but reversed in the CFA + 3-NP group. Besides, ROS levels in the motor cortex of the CFA + 3-NP group were lower than in the Vehicle group. Data are shown for both regions: Vehicle (*n* = 13 images from 4 mice), 3-NP (*n* = 15 images from 5 mice), CFA + 3-NP (*n* = 15 images from 5 mice), and CFA-only (*n* = 13 images from 4 mice). One full-field image per region (motor cortex and striatum) was acquired from 3–4 sections/mouse; each dot represents one image’s mean gray value. The data are expressed as mean ± SEM. One-way ANOVA was performed, followed by Tukey’s post hoc test for multiple comparisons for C and D. Significance level depicted as ^***^p < 0.001, ^**^p < 0.01, and ^*^p < 0.05, scale bar = 100 μm

Although ROS is crucial for cellular machinery, the aberrant elevation of ROS can induce oxidative stress and cellular harm, resulting in DNA damage and cell death (Stefanatos and Sanz 2018). To further explore the neuroprotective potentials of CFA against 3-NP-induced neuronal loss and apoptosis in the motor cortex and striatum, we performed DHE staining to determine the level of ROS generation. Our results revealed 3-NP elevated ROS levels in the motor cortex (*p* < 0.01, Fig. 5A, C) and striatum (*p* < 0.01, Fig. 5B, D) compared to the Vehicle. In line with our hypothesis, CFA significantly decreased ROS levels in the motor cortex (*F*_(3,50)_ = 20.03, *p* < 0.001; Fig. 5C) and striatum (*F*_(3,52)_ = 16.36, *p* < 0.001; Fig. 5D) including a significant reduction between the Vehicle and CFA + 3-NP groups in the motor cortex (*p* < 0.05, Fig. 5C), suggesting the anti-oxidative properties of CFA.

### CFA increased PKM2, a key mitochondrial regulatory cellular protein

**Fig. 6:**
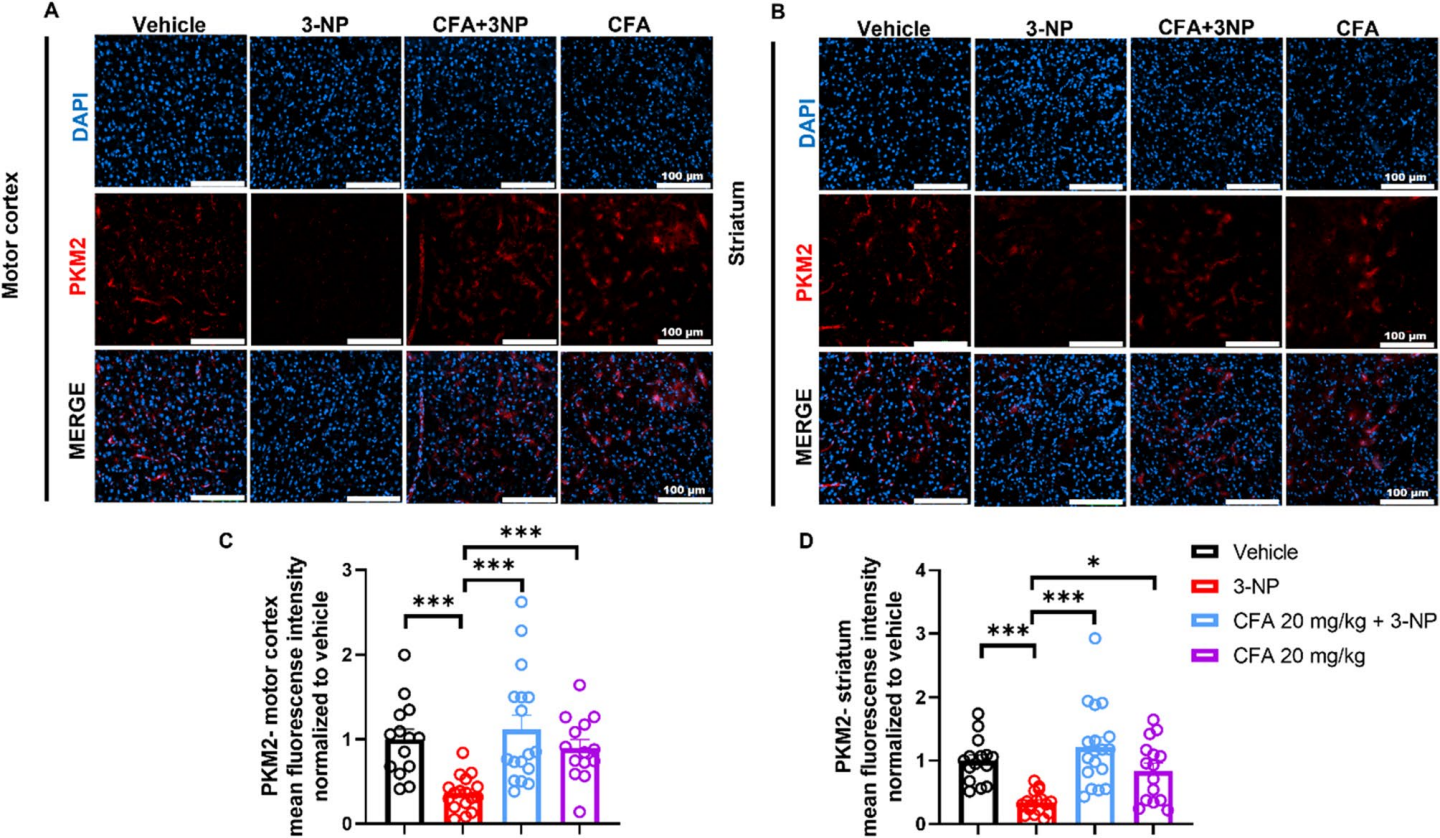
CFA enhanced PKM2 in the 3-NP HD mice. Immunofluorescence images show that 3-NP caused a decrease in **A**, **C** motor cortex and **B**, **D** striatum PKM2 expression, compared to the vehicle and CFA-only groups. Concurrently, it was reversed in the CFA + 3-NP group. Data are shown for: Motor cortex: Vehicle (*n* = 14 images from 4 mice), 3-NP (*n* = 17 images from 5 mice), CFA + 3-NP (*n* = 17 images from 5 mice), CFA-only (*n* = 14 images from 4 mice). Striatum: Vehicle (*n* = 15 images from 4 mice), 3-NP (*n* = 17 images from 5 mice), CFA + 3-NP (*n* = 18 images from 5 mice), CFA-only (*n* = 15 images from 4 mice), from 3–4 sections/mouse. Each dot represents one full-field image’s mean gray value. The data are expressed as mean ± SEM. **C** Kruskal-Wallis test, followed by Dunn’s post hoc test, and **D** One-way ANOVA, followed by Tukey’s post hoc test, were performed for multiple comparisons. Significance level depicted as ***p < 0.001 *p < 0.05, scale bar = 100 μm

As an irreversible inhibitor of succinate dehydrogenase (complex II), 3-NP disrupts both the tricarboxylic acid (TCA) cycle and the electron transport chain, leading to impaired oxidative phosphorylation, ATP depletion, and excessive ROS production, hallmarks of mitochondrial dysfunction and neurodegeneration (Brouillet et al. 2005). PKM2, a key regulator of the glycolytic flux and mitochondrial respiration, is central in determining cellular energy balance (He et al. 2025). It has been reported that increased intracellular reactive oxygen species (ROS) caused inhibition of PKM2 activity (Guo et al. 2016). Therefore, we speculate that the modified expression and function of PKM2 are associated with ROS production, neuronal loss, and apoptosis (Han et al. 2021). Next, we aimed to determine the potential effect of CFA on PKM2 expression in the motor cortex and striatum under 3-NP-induced neurotoxicity using immunofluorescence labeling. Our results showed that 3-NP significantly decreased PKM2 expression in the motor cortex (Fig. 6A and C) and striatum (Fig. 6B and D) compared to the Vehicle (*p* < 0.001) and CFA-only groups (*p* < 0.001 for Fig. 6C; *p* < 0.05 for Fig. 6D). Interestingly, CFA administration significantly increased PKM2 expression in the motor cortex (*H*_(3)_ = 26.06, *p* < 0.001; Fig. 6C) and striatum (*F*_(3,36)_ = 12.20, *p* < 0.001; Fig. 6D) of the 3-NP mice. CFA’s ability to modulate PKM2 expression suggests a mechanism by which it may restore metabolic homeostasis, attenuate ROS generation, and inhibit apoptosis, thereby conferring neuroprotective effects in HD mice’s motor cortex and striatum.

### CFA’s effects on GFAP and IL-1β/IL-6 in 3-NP-HD mice

**Fig. 7:**
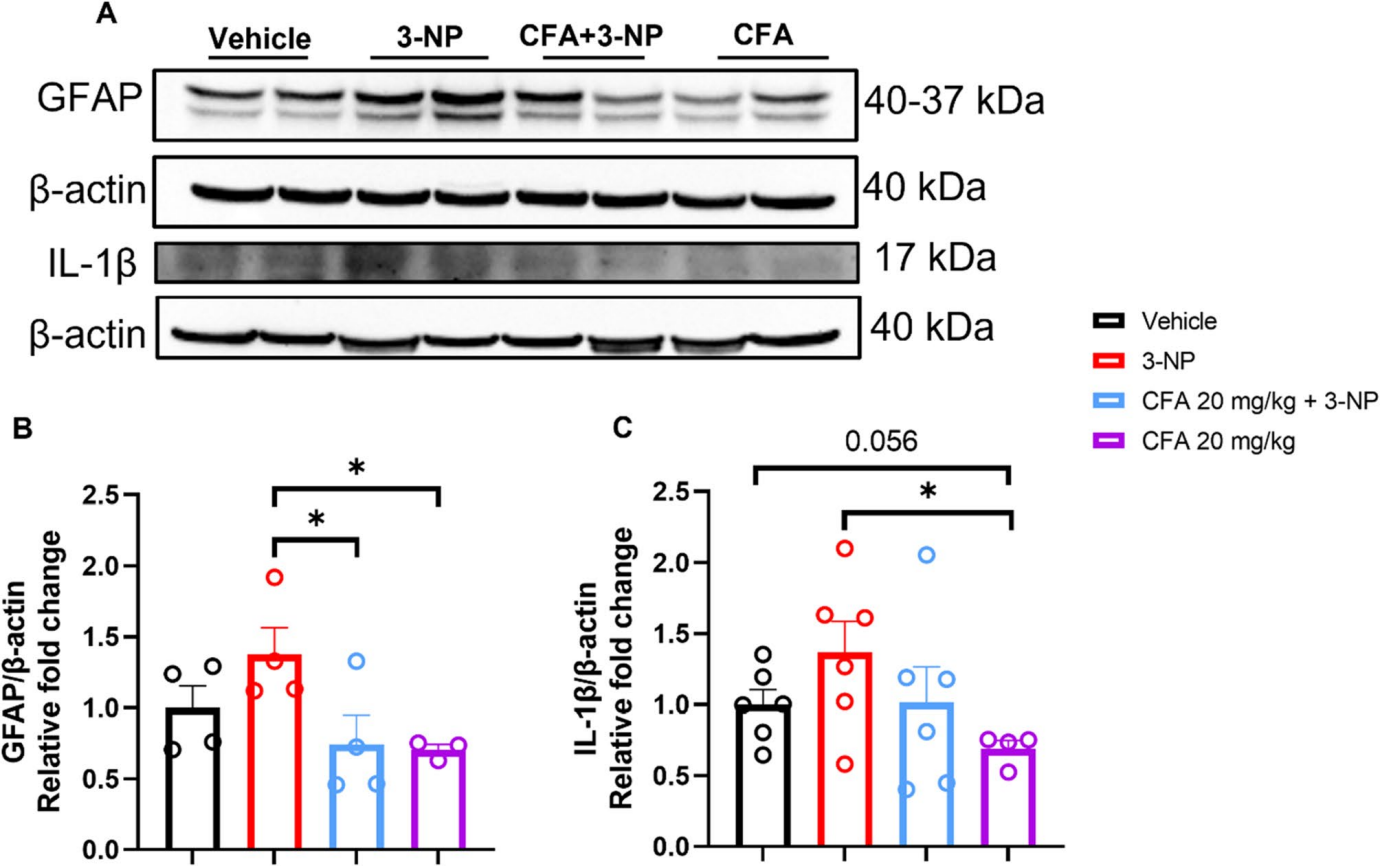
CFA modulates GFAP and IL-1β expression. **A** Representative Western blot images showing the effect of CFA on GFAP and IL-1β in the striatum of 3-NP-HD mice. **B** The GFAP expression bar chart illustrates the significant differences between the 3-NP, CFA + 3-NP, and CFA-only groups. **C** CFA-only decreased IL-1β compared to the 3-NP group. The data are expressed as mean ± SEM. GFAP, *n* = 3–4/group and IL-1β, *n* = 4–6/group. One-way ANOVA testing was performed for **B**, followed by Fisher’s LSD post hoc test for multiple comparisons. An unpaired t-test was applied for **C**. The significance level is depicted as *p < 0.05

In activated astrocytes, the glycolysis pathway is significantly altered (Xiong et al. 2022), triggering the secretion of pro-inflammatory cytokines IL-1β and IL-6. This accelerates ROS generation, ultimately leading to the death of cortical and striatal neurons in HD (Stefanatos and Sanz 2018). Then, we utilized the western blotting assay and immunohistochemical staining to unravel the effect of CFA treatment on 3-NP-induced astrocyte activation and inflammatory responses in the striatum. The results showed that 3-NP administration trend to increase striatal astrocyte activity (*F*_(3,11)_ = 3.221, *p* = 0.065; Fig. 7A). Notably, CFA treatment with or without 3-NP administration significantly decreased the expression level of the glial fibrillary acid protein marker for astrocytes compared to the 3-NP group (*p* < 0.05; Fig. 7B). It suggests the potential efficacy of CFA in maintaining homeostatic levels of astrocytes.

Additionally, we observed a significant trend toward an increase in IL-1β levels in the 3-NP group compared to the CFA-only group (*t*_(8)_ = 2.464, *p* < 0.05; Fig. 7C) and a non-significant downward trend compared to the Vehicle group (*t*_(8)_ = 2.232, *p* = 0.056). However, the CFA + 3-NP group has no significant reduction in the level of IL-1β compared to the 3-NP group.

**Fig. 8:**
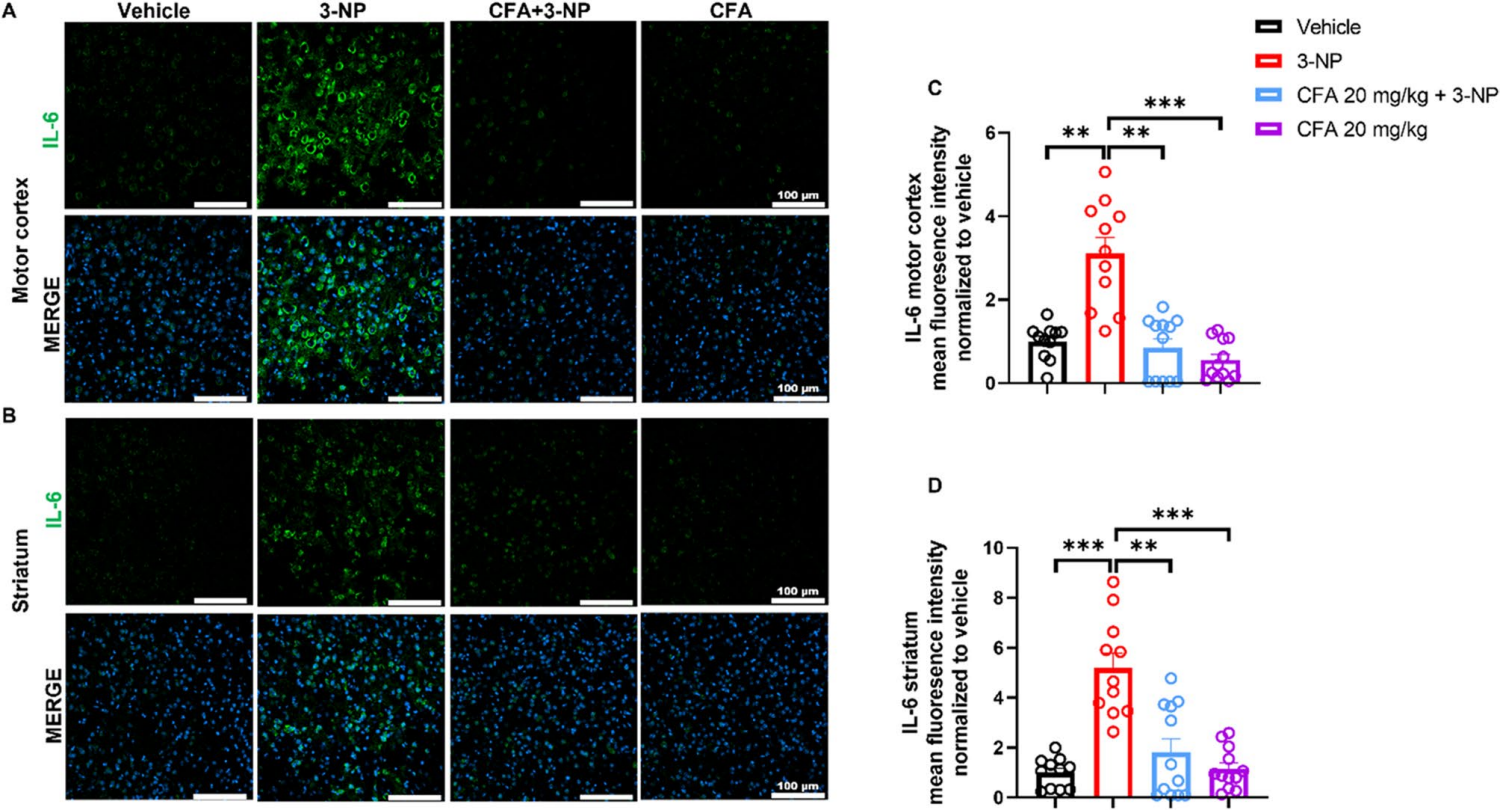
CFA decreased the 3-NP-induced upregulation of IL-6, an inflammatory cytokine. Immunofluorescence images of **A** motor cortex and **B** striatum across groups show increased IL-6 signal in the 3-NP mice compared to the Vehicle and CFA-only groups, but attenuated in the CFA + 3-NP group. Data are shown for both regions: Vehicle (*n* = 11 images from 3 mice), 3-NP (*n* = 11 images from 3 mice), CFA + 3-NP (*n* = 12 images from 3 mice), and CFA-only (*n* = 12 images from 3 mice). One full-field image per region (motor cortex and striatum) was acquired from 3–4 sections/mouse; each dot represents one image’s mean gray value. The data are expressed as mean ± SEM. Kruskal-Wallis test was performed, followed by Dunn’s post hoc test for multiple comparisons. Significance level depicted as ^***^p < 0.001, ^**^p < 0.01, scale bar = 100 μm

The immunohistochemistry images and their quantitative results in the motor cortex (Fig. 8A) and striatum (Fig. 8B) of the 3-NP-injected mice showed significantly elevated IL-6 expression compared to the Vehicle (*p* < 0.01 for Fig. 8C; *p* < 0.001 for Fig. 8D) and CFA-only mice (*p* < 0.001). Remarkably, CFA + 3-NP significantly reduced IL-6 expression compared to the 3-NP group in the motor cortex (*H*_(3)_ = 23.42, *p* < 0.001; Fig. 8C) and striatum (*H*_(3)_ = 20.63, *p* < 0.001; Fig. 8D). The western blot analysis of IL-6 is shown in Supplementary Fig. S8. These results demonstrate the anti-inflammatory benefits of CFA in suppressing excessive inflammatory responses in the 3-NP-injected mice.

### Molecular docking shows that CFA binds to STAT3

We explored the signaling pathway through which CFA exerts its neuroprotective effect using molecular docking. CFA is a phenolic compound; previous research suggested that phenolic compounds modulate JAK2/STAT1 (Akram et al. 2016). We thus hypothesized that CFA might bind to STAT3. Initially, we selected the 1BG1 protein structure as the receptor and CFA as the ligand. Then, we analyzed the interaction using energy binding scores, residues, and atoms.

**Fig. 9:**
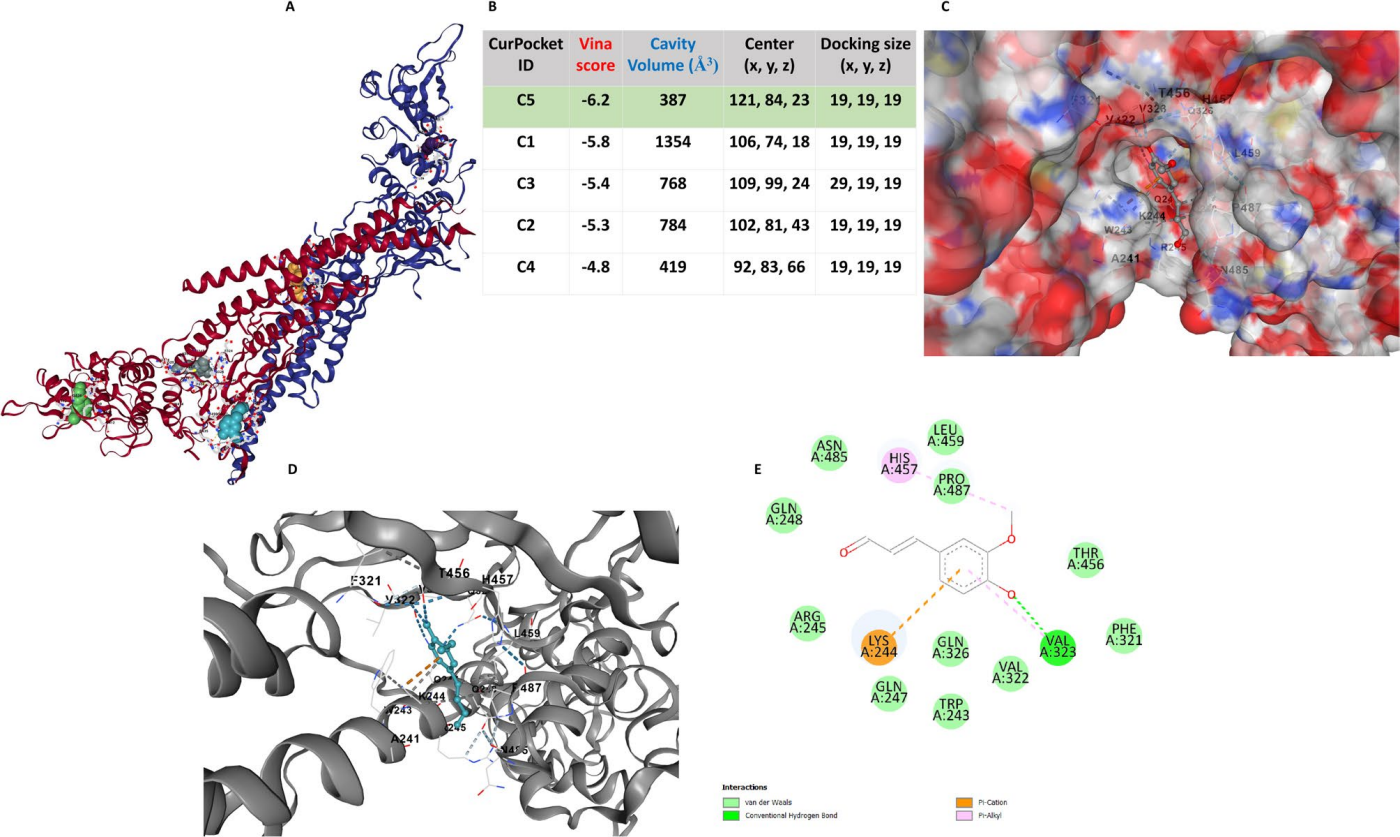
In silico modeled structure cartoon representation of docked complex STAT3/CFA. **A**, **B** CB-DOCK2, cavity blind docking poses (Ludolph et al. 1991), **A** cartoon, **B** Curpocket, and Autodock Vina scores. **C** Electrostatic surface representation of CFA docked within the cavity of STAT3, RED: negatively charged regions; BLUE: positively charged regions; WHITE: neutral or hydrophobic regions. **D** Spherical cartoon and STAT3-helices (gray) with CFA (teal) represent the binding pocket cavity interaction between STAT3 residue and CFA. **E** 2D structure of C5 STAT3 with CFA showing interacting amino acid residues

To investigate the binding pockets and ligand affinity, we used CB-DOCK2, which identified five curpockets (cp.), the top binding cp. C5 with a score of −6.2 kcal/mol and a cavity volume of 387 Å³ (Fig. 9A, B). The electrostatic surface representation reveals the red areas, which indicate negatively charged regions; the blue areas, which indicate positively charged regions; and the white areas, which represent neutral or hydrophobic regions (Fig. 9C). We further show the ribbon representation (gray), displaying the secondary structure (helices and loops) to identify the binding pockets where CFA (teal) interacts (Fig. 9D). Our analysis of the C5 protein-ligand interactions and the receptor-ligand interaction on the 2D diagram revealed contact residues, as shown in (Fig. 9E). We also displayed the 2D diagrams of other binding pockets and their residues (Supplementary Fig. S9). Supplementary Table 5 shows the residues in the binding pockets. We classified the interactions based on 1BG1 structural domains: cp1 (DNA binding domain (DBD) and SH2 domain), cp2 (DBD, SH2 domain, and Transactivation domain (TAD)), cp3 (DBD), cp4 (TAD), and cp5 (DBD and SH2 domain) as shown in (supplementary Table 6). These findings suggest that CFA may inhibit p-STAT3, supporting its potential as an effective inhibitor.

### CFA modulates JAK2/pSTAT3 signaling

**Fig. 10:**
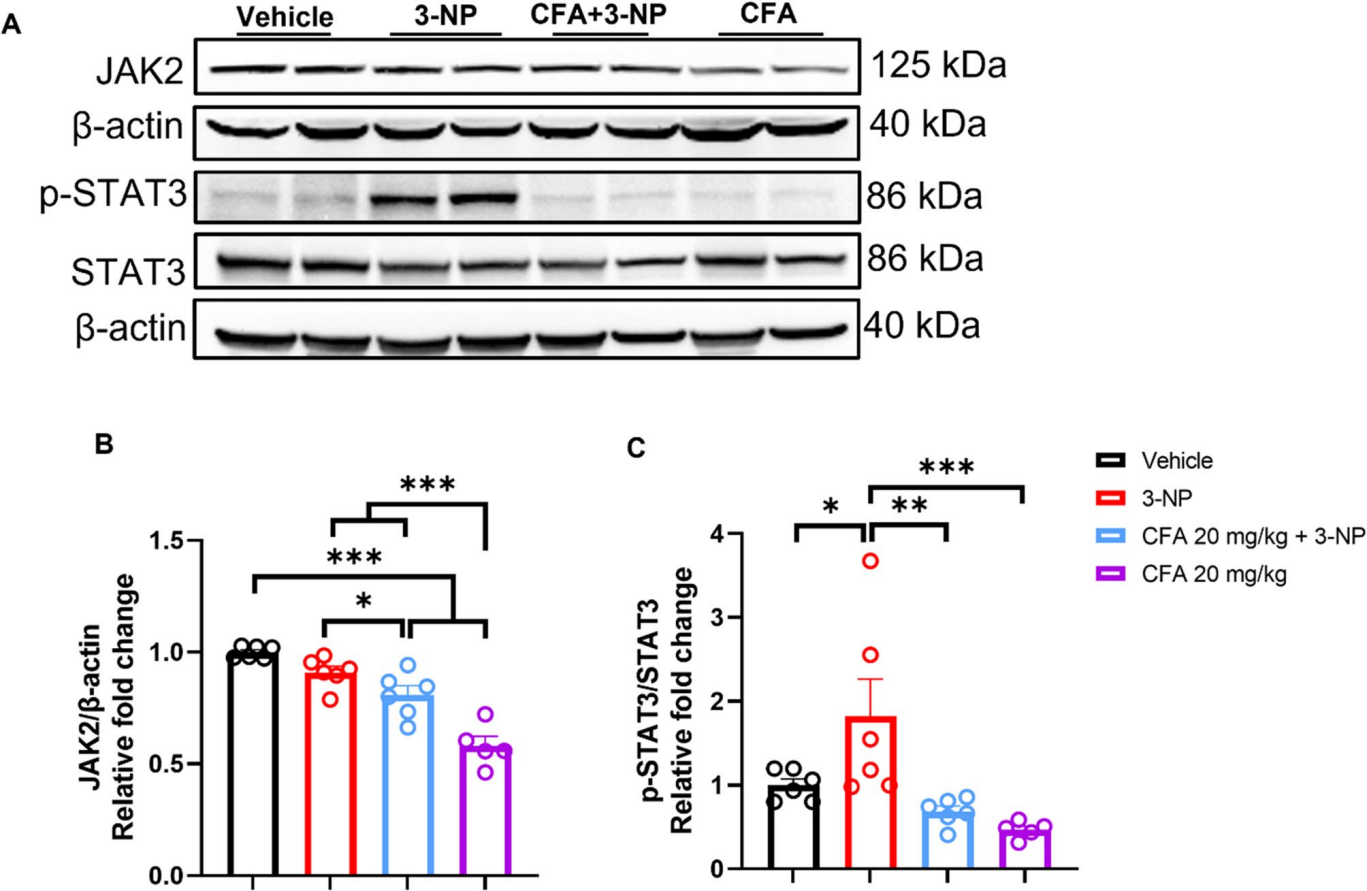
Modulatory effect of CFA on the JAK2/STAT3 signaling.** A** The representative blots for JAK2, p-STAT3, and STAT3 in the striatum across groups. **B** CFA administration with or without 3-NP decreased JAK2 expression compared to the 3-NP and Vehicle groups, while CFA-only decreased JAK2 levels compared to the CFA + 3-NP group. **C** The ratio of STAT3 phosphorylation shows that 3-NP caused elevated p-STAT3/STAT3 compared to Vehicle and CFA-only, but reversed in the CFA + 3-NP group. The data are expressed as mean ± SEM, *n* = 5–6/group. A one-way ANOVA test was performed, followed by Fisher’s LSD post hoc test for multiple comparisons. The significance level is depicted as ***p < 0.001, **p < 0.01, and *p < 0.05

Our molecular Docking showed that CFA could bind to STAT3, so we hypothesized that CFA may attenuate elevated IL-6/JAK2/STAT3 signaling pathways involved in astrocyte reactivity (Ben Haim et al. 2015; Kooshki et al. 2023) in the striatum of the 3-NP mice. Therefore, we utilized a western blot analysis (Fig. 10A) to determine CFA’s effect on JAK2/STAT3 expression in the 3-NP-treated mice. Our findings reveal that CFA-only decreased JAK2 expression (*F*_(3,19)_ = 29.82, *p* < 0.001; Fig. 10B) compared to the 3-NP (*p* < 0.001), Vehicle (*p* < 0.001), and CFA + 3-NP groups (*p* < 0.001). The CFA + 3-NP group also decreased JAK2 levels compared to the Vehicle group (*p* < 0.001) and 3-NP group (*p* < 0.05), while no significant difference was shown in the 3-NP group compared to the Vehicle group (*p* = 0.054).

To validate the molecular docking, we checked the expression of p-STAT3/STAT3. Figure 10A and C show that 3-NP administration significantly upregulated the ratio of p-STAT3/STAT3 levels (*F*_(3,19)_ = 6.229, *p* < 0.01; Fig. 10C) in the striatal samples compared to the Vehicle (*p* < 0.05) and CFA-only (*p* < 0.001). Interestingly, CFA + 3-NP treatment significantly reduced the ratio of p-STAT3/STAT3 compared to the 3-NP group (*p* < 0.01). These results suggest that CFA may exert its neuroprotective and anti-inflammatory impact via the JAK2/STAT3 signaling pathway.

## Discussion

Naturally occurring compounds with pleiotropic activities offer an attractive strategy for disease modification. In this study, we investigated CFA, a phenolic compound derived from various dietary and medicinal sources, with potent anti-oxidative and anti-inflammatory properties (Itoh et al. 2012; Zhang et al. 2016; Pan et al. 2024), for its potential to mitigate HD pathology in a 3-nitropropionic acid (3-NP)–induced HD model.

In this study, intraperitoneal administration of CFA improved neurological deficits and motor coordination, attenuated oxidative stress and apoptosis, and exhibited partial anti-inflammatory effects. Behavioral symptoms such as neurological impairments and motor deficits are indicators of disease severity in HD (Fernagut et al. 2002; Chang et al. 2018). Our study reveals that CFA treatment reversed the neurological, locomotor, and motor deficits in the 3-NP-HD mice model by alleviating the neurological phenotypes and improving locomotor activity and fall latency.

Nissl granules consist of the rough endoplasmic reticulum and ribosomes within neurons, serving as the sites for protein synthesis (Bhati et al. 2023). Exposure to 3-NP disrupts mitochondrial function and energy metabolism, which can damage these structures in a process often observed as chromatolysis or the dissolution of Nissl granules, leading to neuronal death (Bhati et al. 2023; Palanca et al. 2014). Previous studies have reported disruption in the Nissl granules following exposure to 3-NP toxin (Kim et al. 2017a; Jang et al. 2014). In this study, CFA protected the damage of cortical and striatal Nissl bodies, as evidenced by the Nissl staining.

While 3-NP causes neuronal apoptosis (Jang et al. 2014), CFA prevents apoptosis by inhibiting LPS-induced apoptosis in RAW264.7 macrophage cells (Kim et al. 2016). In parallel, our study shows that CFA decreased the apoptotic TUNEL signal in the 3-NP-HD mice.

ROS activation upsets homeostatic energy in brain cells, leading to behavioral dysfunction, energy deficits, and cell death (Stefanatos and Sanz 2018; Sharma et al. 2021). In LPS-activated BV2 microglia, CFA reduced intracellular ROS levels and oxidative damage markers, such as 4-hydroxynonenal (4HNE) and 8-hydroxydeoxyguanosine (8OHdG), via the AMPK/Nrf2 pathway (Park et al. 2024).

At the molecular level, CFA modulates the expression of pyruvate kinase M2 (PKM2**)** - a key metabolic enzyme in the glycolytic pathway and inhibits the phosphorylation of STAT3, a critical transcription factor involved in neuroinflammation and glial activation. PKM2 has been reported to be crucial in regulating neuronal energy metabolism (He et al. 2025). Furthermore, the dual role of PKM2 in regulating glycolysis and motor skill recovery has been documented in models of cerebral ischemia and neurodegeneration (Chen et al. 2018). A study demonstrated that PKM2 deletion caused a significant increase in ROS-induced neuronal damage, progressive loss of dopaminergic neurons, marked reduction of antioxidants, reduced glutathione, and reduced ascorbic acid in adult mice neuronal somata (Wei et al. 2023). This suggests that PKM2 is required for neuronal survival against oxidative damage.

Under severe toxicity, 3–NP decreases ATP and causes mitochondrial membrane depolarization (Liu et al. 2018; Kim et al. 2005), culminating in ROS-mediated oxidation of PKM2 on cellular metabolism and redox balance (Guo et al. 2016). This limits the compensatory glycolytic response and exacerbates energy deficits. Consistently, 3-NP increased ROS and reduced PKM2 levels in our 3-NP-HD model. Interestingly, CFA treatment abated ROS activation and increased PKM2, which may facilitate improved glycolytic flux and ATP production, thereby supporting neuronal survival and function (Adanyeguh et al. 2015). This study provides novel insight into restoring metabolic homeostasis in the HD brain, complementing existing reports on PKM2’s protective role in maintaining neuronal bioenergetics. It is the first to show that CFA reduces 3-NP-induced toxicity in the motor cortex and striatum by modulating oxidative and PKM2-metabolic pathways.

Studies indicate that CFA is an Nrf2 anti-oxidative agonist (Dong et al. 2020; Kim et al. 2016; Chen et al. 2015) and PKM2 modulator in an Alzheimer’s disease model (Dong et al. 2020), depicting its anti-oxidative and metabolic properties. In APP/PS1 AD mice, CFA restored mitochondrial function, ameliorated learning and memory deficits, and potently activated Nrf2 signaling, which was reduced in AD patients and these mice (Dong et al. 2020).

The JAK2/STAT3 signaling cascade mediates neuroinflammation and neuronal injury in HD (Ben Haim et al. 2015; Panda et al. 2024). A study reported that CFA exerts anti-inflammatory properties by selectively inhibiting JAK2/STAT1 signaling and downregulating nitric oxide and iNOS production (Akram et al. 2016). Our molecular docking studies indicate that CFA binds to and inhibits STAT3 activation, potentially reducing the transcription of proinflammatory cytokines. 3-NP inhibits mitochondrial complex II in striatal neurons (Brouillet et al. 2005), triggering ROS (Tunez et al. 2010) and an IL-6/IL-1β cytokine surge (Kim et al. 2017a; Jang et al. 2014) via JAK2/STAT3 in glia (Kim et al. 2017a; Ben Haim et al. 2015). Our in-silico studies show that CFA binds STAT3 at multiple domains (SH2, DBD, and TAD; Supplementary Tables S5 and S6), suggesting it blocks Tyr705 phosphorylation, nuclear import, and transcriptional activity. This triple blockade may interrupt the glial cytokine loop, reducing IL-6/IL-1β and glial activation. This in silico finding was supported by western blot analysis, which revealed suppressed JAK2/STAT3 signaling and a decreased p-STAT3/STAT3 ratio in the striatum. Although our study offers novel insights into CFA’s neuroprotective potential in this model, we did not identify which cell type(s) mediate these effects. This represents a limitation, and future work should delineate the specific cellular mechanisms involved.

Mechanistically, CFA may modulate multiple brain cell types: previous studies suggest that it inhibits TAK1-NF-κB and JAK2-STAT1 signaling in microglia (Park et al. 2024) and leptomeningeal cells (Wang et al. 2020), and activates ERK-driven neurite outgrowth in neurons (Jeon et al. 2019). This multi-cell-type engagement aligns with the oxidative-inflammatory-metabolic triad implicated in HD and may explain the broad histological and behavioral protection observed in our study.

Astrocytes amplify the inflammatory response by microglia in the presence of 3-NP insult, leading to the aberrant release of pro-inflammatory cytokines such as IL-6 and IL-1β (Noureldeen et al. 2024). Moreover, in the LPS-stimulated BV2 microglia, CFA inhibited nitric oxide production and suppressed TNFα, IL-1β, and IL-6 release by targeting the TAK1/MAPK/NFκB signaling cascade (Park et al. 2024). Similar anti-inflammatory effects have been reported for other natural compounds that decreased GFAP activation linked with motor disturbances (Mu et al. 2016) and reduced IL-6 and IL-1β production by inhibiting JAK2/STAT3 signaling pathways in RAW 264.7 cells (Kim et al. 2017b).

Our results show an upward trend between the vehicle and 3-NP GFAP expression, but it is not significant. However, CFA significantly modulates GFAP expression following 3-NP-induced striatal degeneration. This may be due, in part, to the modulatory effect of CFA on STAT3 activity. STAT3 has been known to play a key role in modulating astrocyte activation (O’Callaghan et al. 2014). Herein, independent of the 3-NP effect, CFA treatment modulates STAT3 activity, which in turn impacts GFAP activation as indicated by GFAP protein expression. On the other hand, while immunostaining revealed 3-NP induced elevated IL-6 in the treated mice compared to vehicle, CFA + 3-NP, and CFA-only groups, unexpectedly, in the western blot analysis, 3-NP alone did not elevate striatal IL-6 (Supplementary Fig S8), similarly for IL-1β relative to vehicle. However, treatment with CFA-only significantly reduced IL-6 and showed a non-significant trend toward reduction in IL-1β levels. CFA + 3-NP showed a downward trend in IL-6 and IL-1β levels compared to 3-NP, suggesting partial modulation, though not statistically significant. For IL-6, whole tissue biochemical analysis did not detect significant differences in soluble IL-6 levels. This observed contrast may reflect methodological sensitivities: immunostaining captures localized neuroinflammation in specific cell populations, whereas Western blot quantifies aggregate IL-6 protein across homogenized tissue compartments (Li et al. 2020). 3-NP’s focal neuroinflammatory damage may be concentrated in the area, detected by the staining, but diluted in whole-tissue assays. In part, this is a limitation of this study. Further biochemical assays may require sub-region dissection or concentrated microdialysates to match staining sensitivity. This highlights the need for a multimodal assessment in neurodegenerative diseases for translational purposes. Besides, CFA inhibited the expression of proinflammatory cytokines in LPS-treated mice (Park et al. 2024). Together, this suggests the potential anti-inflammatory effect of CFA in this model.

It is essential to note that the current study employed an acute 3-NP model, which may not fully represent HD’s chronic and progressive nature. While the 60-60-80-80 mg/kg 3-NP regimen is effective at rapidly inducing reproducible striatal damage and neurological deficits for the study of neurotoxicity and testing acute neuroprotective interventions (Kim et al. 2017a; Jang et al. 2014; Huang et al. 2006), its acute nature results in a different lesion pattern (diffuse versus selective lateral striatal loss) (Tunez et al. 2010; Brouillet 2014; Brouillet et al. 1999), a different type of insult (rapid, massive cell death versus sustained metabolic dysfunction), and may not replicate the full behavioral complexity seen in chronic HD or potentially in chronic 3-NP models (Tunez et al. 2010). The specific outcome is also highly dependent on the exact protocol and animal characteristics, such as strain and age (Gabrielson et al. 2001). Future studies are warranted to evaluate the long-term neuroprotective effects of CFA in chronic HD models and the 3-NP peak plasma and brain concentrations for the acute dose regimen.

The preventive and protective effects of CFA in both in vitro, systemic inflammation, and AD animal models suggest that CFA warrants evaluation in HD models, including human subjects, particularly those in early or prodromal stages. A study confirmed CFA’s ability to cross the BBB and promote neurite outgrowth, finding that daily administration of 10 mg/kg for 7 days increased hippocampal dendritic length (Jeon et al. 2019). Its neurite‑promoting action (Jeon et al. 2019), neuron protection, and learning and memory improvement (Dong et al. 2020) may synergize with synaptic plasticity‑oriented interventions in HD investigation.

Supporting CFA’s therapeutic potentials, our in silico analyses using SwissADME and ProTox 3.0 (Supplementary Tables S1, S2, and S3) revealed high gastrointestinal absorption, efficient blood-brain barrier (BBB) penetration (permeability score: 0.80), and low molecular weight (178.18 Da) (Akram et al. 2016). Compared to nutraceuticals like CUR (high CYP inhibition, poor solubility), RES (limited BBB permeability) (Shimazu et al. 2021), CGA (low GI absorption) (Clifford et al. 2020), and a plasma concentration reported to be 22 times higher than that of RES 1.5 h after equimolar i.p. administration to the mice (Radnai et al. 2009), CFA’s balanced lipophilicity (log *P* = 1.56) and selective CYP2C9 interaction (activity score: 0.72) reduce drug-drug interaction risks compared to CUR, which broadly inhibits all major CYPs (CYP2C19 (0.94), CYP2C9 (0.89), CYP3A4 (0.63).

Oral administration in mice (0.2 mmol/kg) confirmed rapid absorption, with peak brain concentrations at 1 h, supporting effective CNS delivery (Dong et al. 2020). CFA’s safety profile indicates that its oral LD^50^ values (300 mg/kg in mice, 980 mg/kg in rats, 3200 mg/kg in rabbits) (Dong et al. 2020) demonstrate its suitability for therapeutic doses in HD models. CFA has regulatory acceptability as a food flavoring (Dong et al. 2020). Oral administration at 100 mg/kg/day for seven consecutive days showed no hepatotoxicity (Chen et al. 2015), and doses up to 6 mg/kg in mice showed no overt toxicity (Park et al. 2024). However, ProTox 3.0 predicted low probabilities of hepatotoxicity (*p* = 0.50) and nephrotoxicity (*p* = 0.51). CFA’s predicted immunotoxicity (0.79) is lower than CUR’s (0.92) and CGA’s (0.99) (Supplementary Table S3), yet still notable. This suggests CFA is well-tolerated at therapeutic doses, though prolonged use warrants monitoring. Although we did not measure CFA’s brain levels in this study, prior reports suggest CFA may reach pharmacologically effective levels at the lesion site (Dong et al. 2020; Park et al. 2024; Jeon et al. 2019). Our in silico pharmacokinetic profiling supports CFA’s drug-likeness, with a synthetic accessibility score of 1.88, indicating it is readily synthesizable, and a parallel bioavailability score of 0.55, to that of CUR and RES (Supplementary Table S2). These properties make CFA a promising candidate for therapeutic applications, overcoming solubility and absorption limitations that hinder many nutraceuticals’ translational potential.

In our study, 3-NP, a Complex II irreversible inhibitor (Tunez et al. 2010), triggers a ROS surge that depletes ATP and suppresses PKM2, predisposing neurons to degeneration. CFA reduced ROS, restoring redox balance. This curbs excessive glial JAK2/STAT3-mediated IL-6/IL-1β release, interrupting cytokine inflammation. With inflammation reduced, PKM2 levels recover, sustaining glycolysis and ATP production to prevent TUNEL-apoptosis. This sequential cascade initiated by ROS neutralization, followed by dampened inflammation and metabolic restoration, preserves neuronal integrity and improves behavior, highlighting ROS suppression as the proximal (and likely causal) event in CFA’s neuroprotective mechanism, mapping onto the principal HD pathogenic axes.

## Conclusion

Our study provides novel evidence that CFA confers neuroprotection in an HD-like model by preserving neuronal integrity, as indicated by Nissl substance retention and partial anti-inflammatory effect, decreased oxidative stress, and apoptosis in the motor cortex and striatum. These protective effects are accompanied by restoring metabolic homeostasis, potentially mediated through modulation of the JAK2/STAT3 signaling pathway and regulation of PKM2 expression. These findings emphasize the translational potential of CFA as a disease-modifying therapeutic for HD pathologies.

## Supplementary Information

Supplementary Material 1.

## Data Availability

This published article and its supplementary information files include all data generated or analyzed during this study. Further inquiries can be directed to the corresponding author.
