# Supplementary material for: Coniferaldehyde reverses 3-nitropropionic acid-induced Huntington’s disease pathologies via PKM2 restoration and JAK2/STAT3 inhibition
Source: Mol Med. 2025 Jul 31;31:271. doi: 10.1186/s10020-025-01308-0 (PMC12312528; doi:10.1186/s10020-025-01308-0)
Supplement: Supplementary file 1 — Supplementary Material 1. [file 10020_2025_1308_MOESM1_ESM.docx]

**Supplementary Material**

Supplemental Title Page – S1

Figure S1. Experimental design and procedure– S2

Figure S2. CFA alleviates 3-NP-induced neurological, motor, and abnormal postural phenotypes– S3

Figure S3. CFA significantly rescued 3-NP-induced locomotor and movement deficit behavior in HD mice – S4

Figure S8. CFA decreased IL-6 in the 3-NP HD mice– S5

Figure S9. In silico modeled structure cartoon representation of docked complex STAT3/CFA–S6

Table S1. SwissADME analysis of CFA- S7

Table S2. SwissADME - Predicted Pharmacokinetic and chemical properties of CFA and curcumin, resveratrol, and chlorogenic acid – S8

Table S3. ProTox 3.0 predicted toxicity profiles of CFA, in comparison to other compounds -S9

Table S4. 3-NP-induced clinical signs and severity score – S10

Table S5. Curpockets ID and contact residue of CFA/STAT3 complex – S11

Table S6. Residue Classification according to STAT3 Domains – S12

**S1**

**Coniferaldehyde reverses 3-nitropropionic acid-induced Huntington’s disease pathologies via PKM2 restoration and JAK2/STAT3 inhibition**

*Ayooluwa Gabriel Ibiayo^1^, Peeraporn Varinthra^1^, Mukundan Nagarajan^2^, Ingrid Y Liu^1*^*

^1^Institute of Medical Sciences, College of Medicine, Tzu Chi University, Hualien City, 97004, Taiwan.

^2^Department of Molecular Biology and Human Genetics, College of Medicine, Tzu Chi University, Hualien City, 97004, Taiwan.

***Corresponding author:**

Ingrid Y Liu,

Professor, Institute of Medical Sciences,

College of Medicine,

Tzu Chi University, Hualien, Taiwan.

Email: [ycliu@mail.tcu.edu.tw](mailto:ycliu@mail.tcu.edu.tw)

**S2**


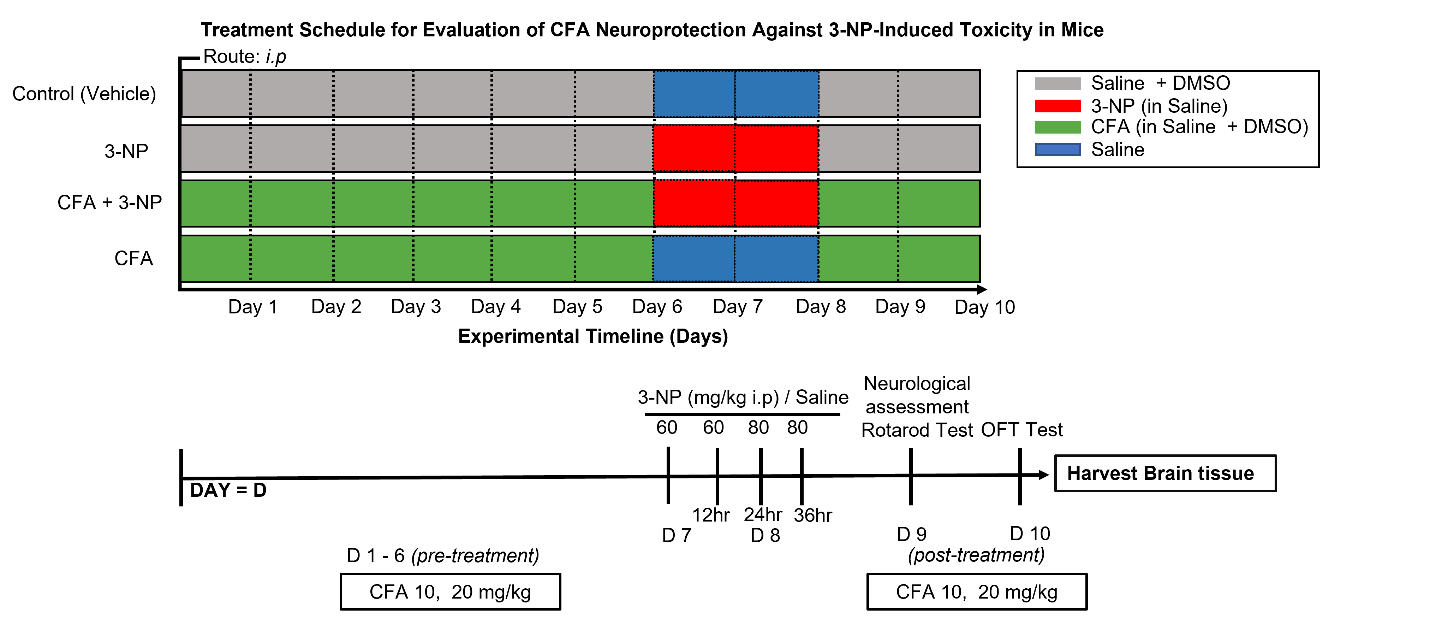


**Supplementary Figure S1. Experimental design and procedure**


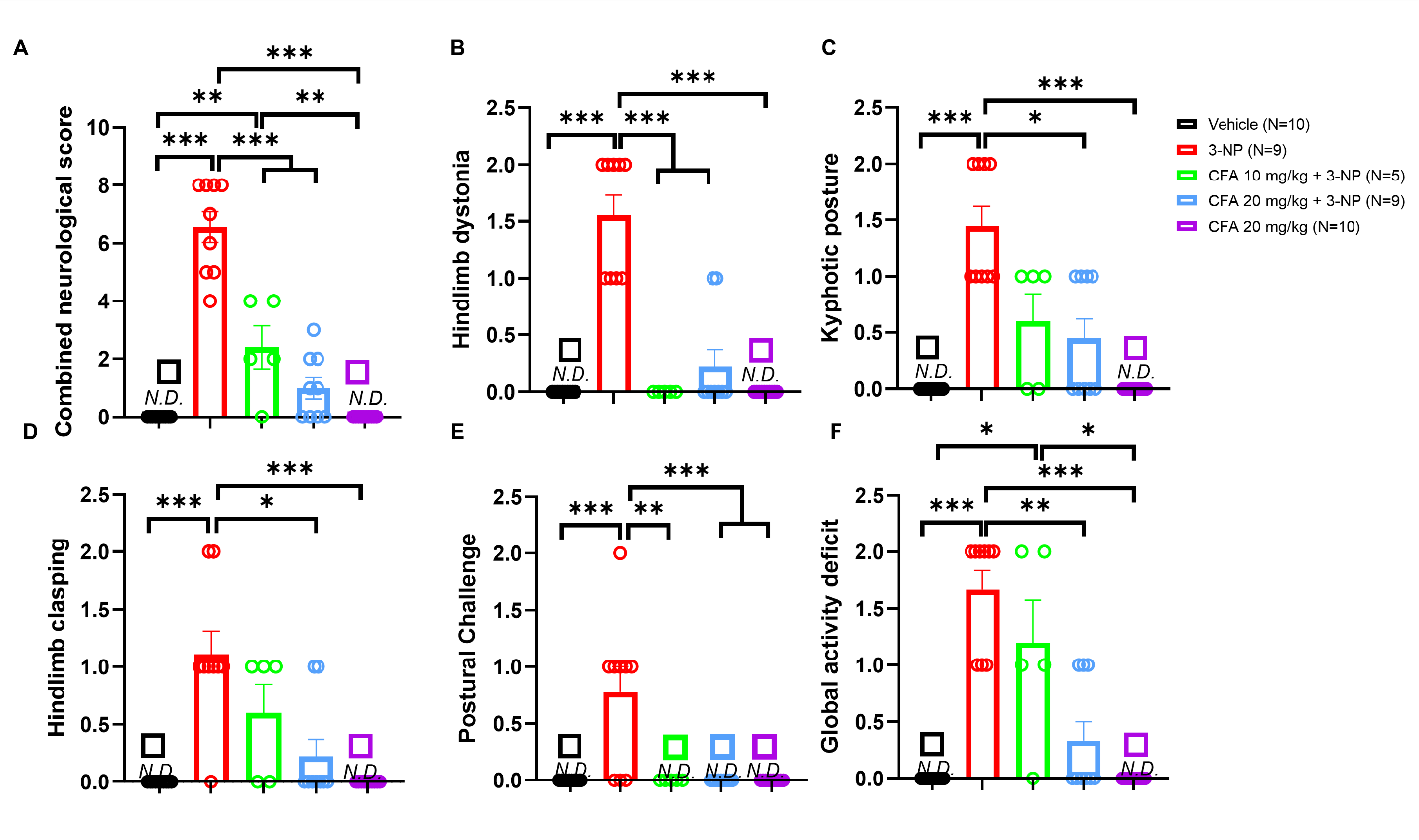
**S3**

**Supplementary Figure S2**. **CFA** **alleviates 3-NP-induced neurological, motor, and abnormal postural phenotypes**. The neurological assessment demonstrated (A) the neurological score, (B) hindlimb dystonia, (C) kyphotic posture, (D) hindlimb clasping, (E) postural challenge, and (F) global activity deficit in a free movement environment significantly impaired in the 3-NP group compared to vehicle and 20 mg/kg CFA only groups but significantly improved in CFA 20 mg/kg + 3-NP group, across all neurological parameters. The CFA 10 mg/kg + 3-NP group shows improvement in (A), (B), and (E). The data are expressed as mean ± SEM. The assessment scale is rated 0 = no detectable neurological deficit (N.D.), 1 = mild deficit, and 2 = severe deficit. One-way ANOVA followed by Tukey’s post hoc test was performed for (A), and Dunn’s post hoc multiple comparisons for (B to F). Significance level depicted as ****p* < 0.001, ***p* < 0.01, and **p* < 0.05. Vehicle, n = 10; 3-NP, n = 9; CFA 10 mg/kg+3NP, n = 5; CFA 20 mg/kg+3NP, n = 9; CFA, n = 10.


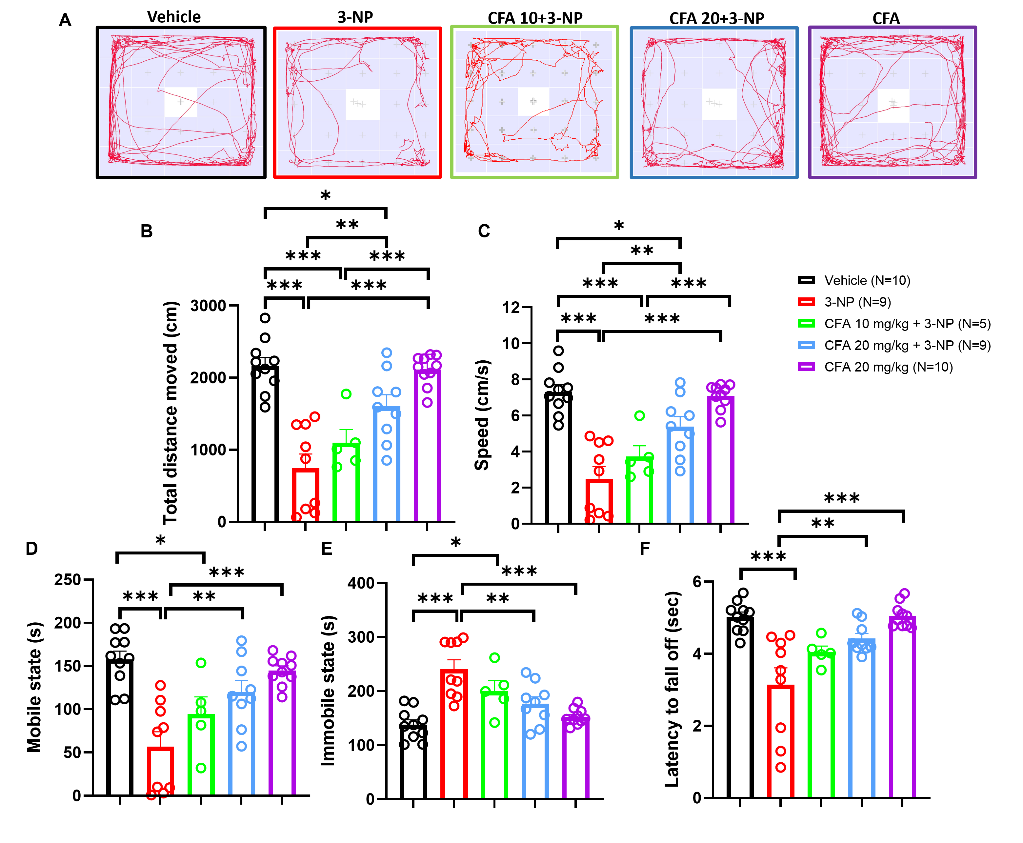
**S4**

**Supplementary Figure S3. CFA significantly rescued 3-NP-induced locomotor and movement deficit behavior in HD mice.** (A) OFT movement tracking images. The 3-NP group showed significantly decreased (B) total distance moved and (C) speed compared to vehicle and CFA-only groups, while significantly rescued after CFA 20 mg/kg administration. CFA 10 mg/kg dose showed no effect. The 3-NP insult reduced (D) mobile state duration and increased (E) immobility state, whereas CFA at 20 mg/kg but not at 10 mg/kg ameliorated these mobility impairments. (F) Rotarod analysis shows that CFA 20 mg/kg treatment abated the motor coordination deficit caused by 3-NP but not at 10 mg/kg. For (F), the data are skewed positively, and a natural log (Y=Ln(Y)) transformation was applied to attain normality. The data are expressed as mean ± SEM. One-way ANOVA followed by Tukey’s post hoc test was performed for B to F. Significance level depicted as ****p* < 0.001 ***p* < 0.01, and **p* < 0.05. Vehicle, *n* = 10; 3-NP, *n* = 9; CFA+3NP, *n* = 9; CFA, *n* = 10.

**S5**


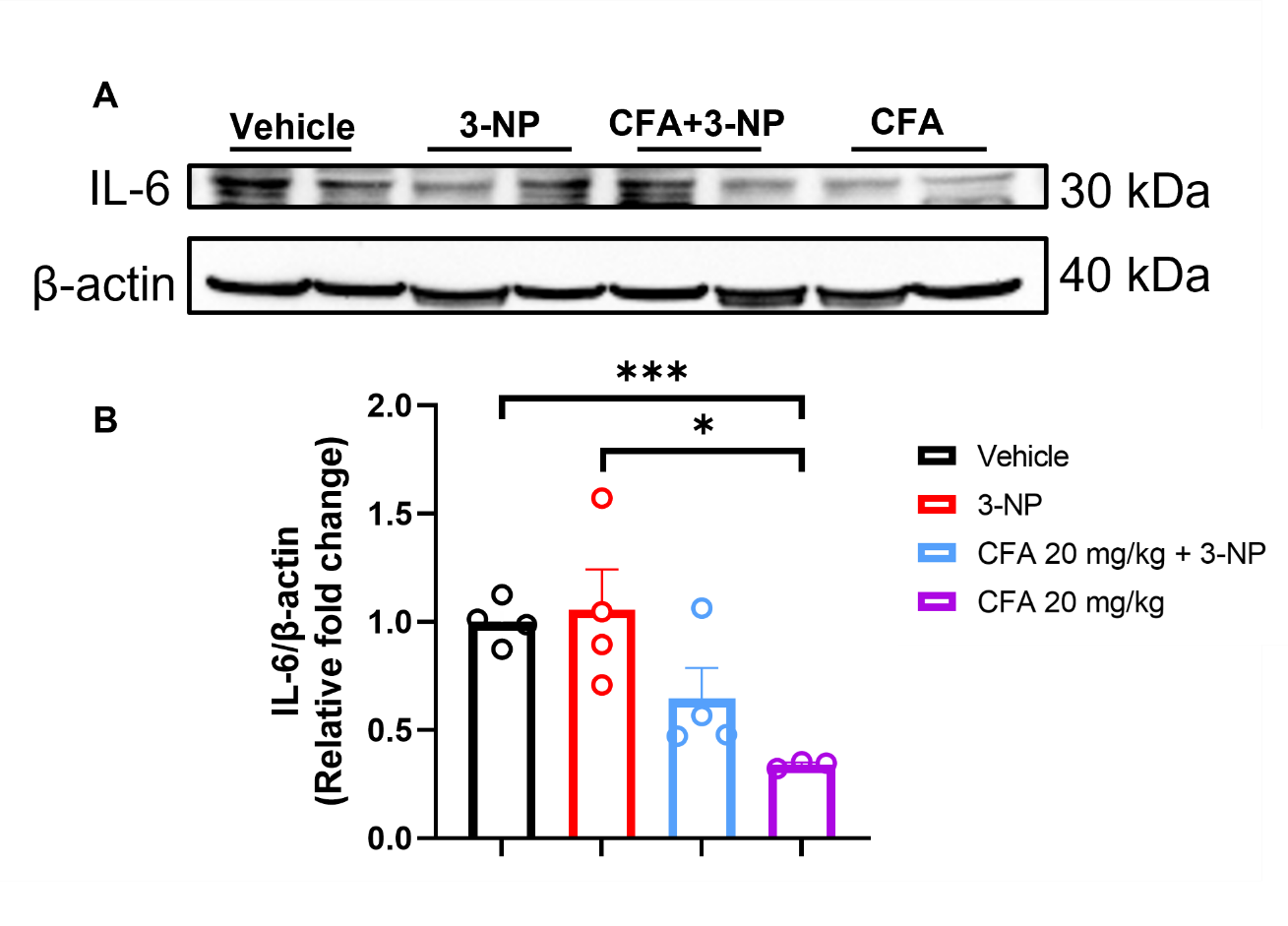


**Supplementary Figure S8. CFA decreased IL-6 in the 3-NP HD mice.** (A) Representative Western blot images showing the effect of CFA on IL-6 in the striatum of 3-NP-HD mice. (B) CFA-only decreased IL-6 level compared to the vehicle and 3-NP group. There is a trend-like decrease in the CFA+3-NP group compared to the 3-NP-only group. The data are expressed as mean ± SEM, *n* = 3 - 4/group. Kruskal-Wallis testing was performed, followed by an unpaired t-test. The significance level is depicted as **p* < 0.05.

**S6**


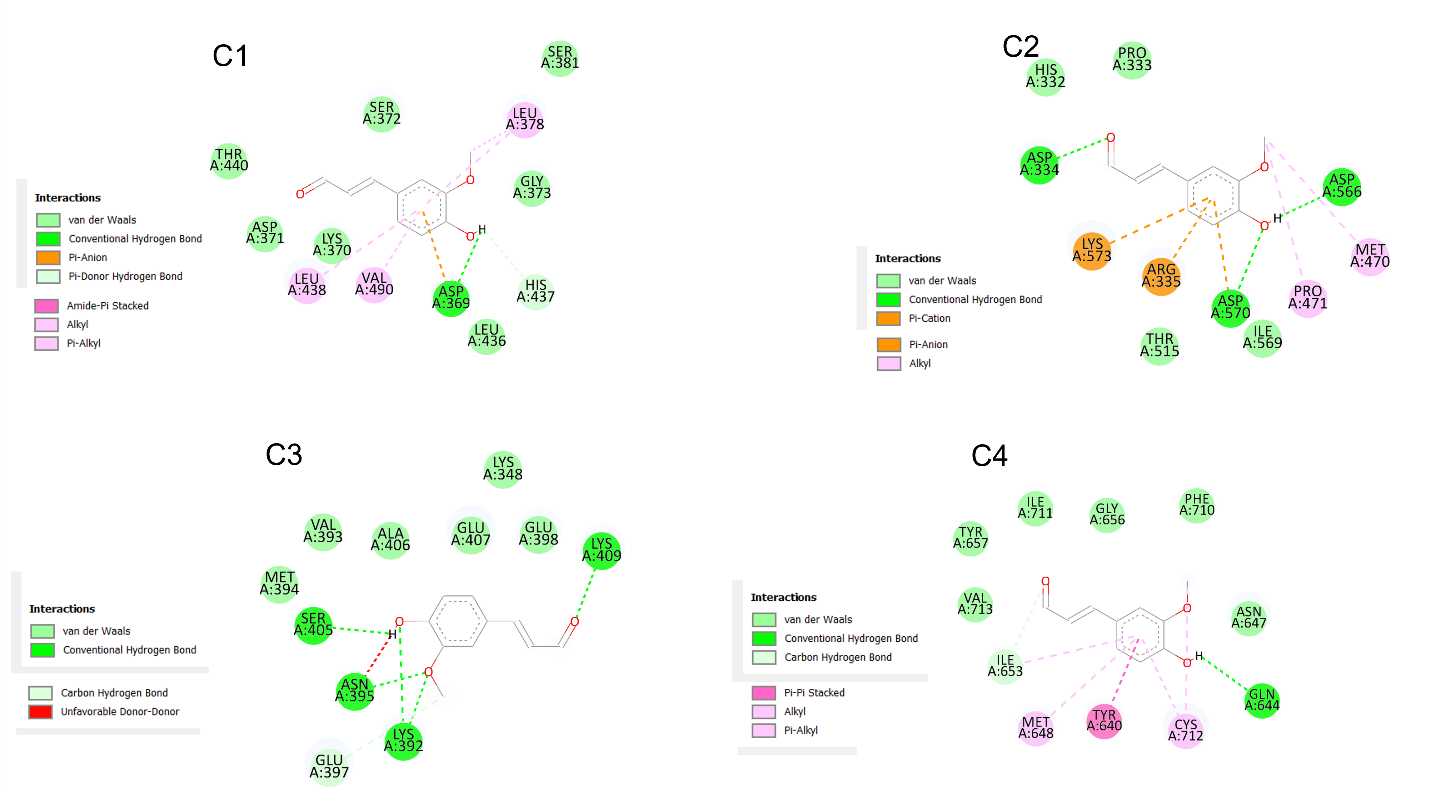


**Supplementary Figure. S9**. **In silico modeled structure cartoon representation of docked complex STAT3/CFA.** CB-DOCK2, cavity blind docking poses (C1-C4), 2D structure of STAT3 with CFA showing interacting amino acid residues.

**S7**

**Supplementary Table S1**. SwissADME Analysis for CFA

| **Property Category** | **Parameter & Value** | **Value** |
| --- | --- | --- |
| Physicochemical Properties | Molecular Weight:  TPSA H-bond Donors H-bond Acceptors Rotatable Bonds | 178.18 g/mol 46.53 Å²  1  3  3 |
| Lipophilicity | Consensus Log P SILICOS-IT Log P | 1.56  2.01 |
| Water Solubility | Log S (SILICOS-IT) Solubility | -1.99  1.84 mg/ml |
| Pharmacokinetics | GI Absorption BBB Permeant P-gp Substrate | High  Yes  No |
| Metabolism | CYP Inhibition: major CYPs (CYP1A2, 2C19, 2C9, 2D6, 3A4) | No |
| Drug-likeness | Lipinski: Yes Bioavailability Score: 0.55 | Yes  0.55 |
| Medicinal Chemistry Alerts | PAINS Brenk  Leadlikeness | 0 alert  2 alerts (aldehyde, michael_acceptor_1)  No; 1 violation: MW<250 |
| Synthetic Accessibility | Score | 1.88 |

**S8**

**Supplementary Table S2.** SwissADME **-** predicted Pharmacokinetic and chemical properties of CFA and curcumin, resveratrol, and chlorogenic acid

| **Parameters** | **Coniferaldehyde** | **Curcumin** | **Resveratrol** | **Chlorogenic acid** |
| --- | --- | --- | --- | --- |
| Molecular weight (MW) | 178.18 | 368.38 | 228.24 | 354.31 |
| TPSA | 46.53 | 93.06 | 60.69 | 164.75 |
| Consensus Log P | 1.56 | 3.03 | 2.48 | -0.39 |
| Silicos-IT class | Soluble | Moderately soluble | Soluble | Soluble |
| GI absorption | High | High | High | Low |
| BBB permeant | Yes | No | Yes | No |
| CYP1A2 inhibitor | No | No | Yes | No |
| CYP2C19 inhibitor | No | No | No | No |
| CYP2C9 inhibitor | No | Yes | Yes | No |
| CYP2D6 inhibitor | No | No | No | No |
| CYP3A4 inhibitor | No | Yes | Yes | No |
| Lipinski #violations | 0 | 0 | 0 | 1 |
| Bioavailability Score | 0.55 | 0.55 | 0.55 | 0.11 |
| Synthetic Accessibility | 1.88 | 2.97 | 2.02 | 4.16 |

**S9**

**Supplementary Table S3.** ProTox 3.0 predicted toxicity profiles of CFA, in comparison to other compounds.

| **Toxicity** | **Coniferaldehyde** | **Curcumin** | **Resveratrol** | **Chlorogenic Acid** |
| --- | --- | --- | --- | --- |
| LD 50 mg/kg | 1560 | 2000 | 1560 | 5000 |
| Class | 4 | 4 | 4 | 5 |
| Hepatotoxicity | Active (0.50) | Inactive (0.61) | Inactive (0.74) | Inactive (0.72) |
| Neurotoxicity | Inactive (0.55) | Inactive (0.81) | Inactive (0.77) | Inactive (0.89) |
| Nephrotoxicity | Active (0.51) | Active (0.59) | Active (0.59) | Active (0.56) |
| Respiratory toxicity | Inactive (0.97) | Inactive (0.77) | Inactive (0.57) | Active (0.57) |
| Cardiotoxicity | Inactive (0.85) | Active (0.55) | Active (0.51) | Inactive (0.99) |
| Carcinogenicity | Inactive (0.63) | Inactive (0.84) | Inactive (0.71) | Inactive (0.68) |
| Immunotoxicity | Active (0.79) | Active (0.92) | Inactive (0.86) | Active (0.99) |
| Mutagenicity | Inactive (0.85) | Inactive (0.88) | Inactive (0.92) | Inactive (0.93) |
| Cytotoxicity | Inactive (0.90) | Inactive (0.88) | Inactive (0.98) | Inactive (0.80) |
| Clinical toxicity | Inactive (0.53) | Active (0.61) | Inactive (0.60) | Active (0.67) |
| Nutritional toxicity | Inactive (0.90) | Inactive (0.74) | Inactive (0.89) | Inactive (0.64) |
| BBB - barrier | Active (0.80) | Active (0.58) | Inactive (0.55) | Active (0.60) |
| CYP1A2 | Inactive (0.78) | Inactive (0.78) | Active (0.92) | Inactive (0.96) |
| CYP2C19 | Inactive (0.66) | Active (0.94) | Inactive (0.71) | Inactive (0.98) |
| CYP2C9 | Active (0.72) | Active (0.89) | Active (0.76) | Inactive (0.57) |
| CYP2D6 | Inactive (0.66) | Inactive (0.81) | Inactive (0.80) | Inactive (0.88) |
| CYP3A4 | Inactive (0.92) | Active (0.63) | Active (1.0) | Inactive (0.99) |
| CYP2E1 | Inactive (1.0) | Inactive (1.0) | Inactive (0.99) | Inactive (1.0) |

These toxicity assessments rely on lethal dose predictions. The probability-value in brackets ‘‘()’’ indicates the likelihood of activity or inactivity. The probability value shown indicates the confidence level of the prediction. Targets with confidence scores below 70% (0.7) are typically excluded and labeled as “Below Threshold.”

**S10**

**Supplementary Table S4**. 3-NP-induced clinical signs and severity score

| **Clinical Sign** | **0 (Normal)** | **1 (Mild Deficit)** | **2 (Severe Deficit)** |
| --- | --- | --- | --- |
| General Activity | Normal activity, regular grooming | Slight reduction in movement (>50% of normal), occasional reduced grooming | Marked reduction or absence of movement, no grooming |
| Hindlimb Dystonia | No hindlimb dystonia | Mild dystonia without gait impairment | Persistent hindlimb dystonia |
| Hindlimb Clasping | No clasping | Intermittent clasping | Hindlimbs clenched against the abdomen |
| Truncal Dystonia (Kyphosis) | No trunk flexion, normal posture | Slight flexion of trunk, mild kyphosis | Pronounced kyphosis with spine curvature |
| Postural Challenge/Recumbency | Stable posture, no recumbency | Moderate postural impairment; falls but recovers quickly | Persistent recumbency, moribund; bilateral hindlimb extension |

**S11 Supplementary Table S5.** Curpockets ID and contact residue of CFA/STAT3 complex

| Cp-ID | Cavities_  volume | center_x | center_y | center_z | size_x | size_y | size_z | score | contact_residue |
| --- | --- | --- | --- | --- | --- | --- | --- | --- | --- |
| 1 | 1354 | 105.911 | 73.993 | 17.835 | 19 | 19 | 19 | -5.8 | CYS:367:A,ILE:368:A,ASP:369:A,LYS:370:A,ASP:371:A,SER:372:A,GLY:373:A,ASP:374:A,VAL:375:A,ALA:377:A,LEU:378:A,ARG:379:A,GLY:380:A,SER:381:A,ARG:382:A,LYS:383:A,PHE:384:A,GLN:416:A,CYS:418:A,ASN:420:A,GLY:421:A,GLY:422:A,LEU:436:A,HIS:437:A,LEU:438:A,ILE:439:A,THR:440:A,GLU:455:A,LYS:488:A,VAL:490:A,ASN:491:A |
| 2 | 784 | 102.37 | 81.277 | 43.142 | 19 | 19 | 19 | -5.3 | MET:331:A,HIS:332:A,PRO:333:A,ASP:334:A,ARG:335:A,ILE:467:A,CYS:468:A,MET:470:A,PRO:471:A,TRP:474:A,THR:515:A,TRP:562:A,ASP:566:A,ASN:567:A,ILE:569:A,ASP:570:A,LYS:573:A,LYS:574:A,GLU:616:A,LYS:642:A |
| 3 | 768 | 109.03 | 99.237 | 23.635 | 29 | 19 | 19 | -5.4 | ILE:258:A,CYS:259:A,LEU:260:A,ASP:261:A,GLU:264:A,GLU:324:A,ARG:325:A,PRO:330:A,GLN:344:A,THR:346:A,LYS:348:A,ARG:350:A,TYR:360:A,LYS:392:A,VAL:393:A,MET:394:A,ASN:395:A,MET:396:A,GLU:397:A,GLU:398:A,ASN:400:A,ASN:401:A,SER:403:A,LEU:404:A,SER:405:A,ALA:406:A,GLU:407:A,LYS:409:A,HIS:410:A |
| 4 | 419 | 91.716 | 83.236 | 66.014 | 19 | 19 | 19 | -4.8 | TRP:623:A,GLU:625:A,LYS:626:A,GLN:635:A,SER:636:A,VAL:637:A,GLU:638:A,PRO:639:A,TYR:640:A,GLN:644:A,LEU:645:A,ASN:647:A,MET:648:A,GLU:652:A,ILE:653:A,GLY:656:A,TYR:657:A,PHE:710:A,ILE:711:A,CYS:712:A,VAL:713:A,THR:714:A,PRO:715:A,PHE:716:A |
| 5 | 387 | 120.665 | 83.982 | 22.569 | 19 | 19 | 19 | -6.2 | ALA:241:A,ASP:242:A,TRP:243:A,LYS:244:A,ARG:245:A,ARG:246:A,GLN:247:A,GLN:248:A,LYS:318:A,SER:319:A,PHE:321:A,VAL:322:A,VAL:323:A,GLU:324:A,GLN:326:A,GLU:455:A,THR:456:A,HIS:457:A,SER:458:A,LEU:459:A,THR:484:A,ASN:485:A,ASN:486:A,PRO:487:A |

**S12**

**Supplementary Table S6.** Residue Classification according to STAT3 Domains

| CP | Residue Range | Domain |
| --- | --- | --- |
| 1 | 367–491 | DBD, SH2 Domain |
| 2 | 331–642 | DBD, SH2 Domain, TAD |
| 3 | 258–410 | DBD |
| 4 | 623–716 | TAD |
| 5 | 241–487 | DBD, SH2 Domain |

In terms of classifying the interaction between CFA and protein residues within the binding cavities of 1bg1, Curpocket (CP)1 (367- 491 DBD and SH2 domain); CP 2 (331 – 642, DBD, SH2 domain and TAD), CP 3 (258 – 410, DBD), CP 4 (623 – 716, TAD), CP 5 (241 – 487, DBD and SH2 domain).

*DBD DNA binding domain; SH2, Src Homology 2 domain; TAD, Transactivation domain*
